# Dietary Protein Consumption and the Risk of Type 2 Diabetes: ADose-Response Meta-Analysis of Prospective Studies

**DOI:** 10.3390/nu11112783

**Published:** 2019-11-15

**Authors:** Mengying Fan, Yuqian Li, Chongjian Wang, Zhenxing Mao, Wen Zhou, Lulu Zhang, Xiu Yang, Songyang Cui, Linlin Li

**Affiliations:** 1 Department of Epidemiology and Health Statistics, College of Public Health, Zhengzhou University, Zhengzhou 450000, China; 15836150920@163.com (M.F.); Tjwcj2005@126.com (C.W.); maozhr@gmail.com (Z.M.); zwzzdx@126.com (W.Z.); lulzhang@126.com (L.Z.); 15727571433@163.com (X.Y.); sycui@foxmail.com (S.C.); 2 Department of Clinical Pharmacology, School of Pharmaceutical Science, Zhengzhou University, Zhengzhou 450000, China; liyuqian0214@126.com

**Keywords:** protein, diet, type 2 diabetes, dose-response, meta-analysis

## Abstract

The relationship between dietary protein consumption and the risk of type 2 diabetes (T2D) has been inconsistent. The aim of this meta-analysis was to explore the relations between dietary protein consumption and the risk of T2D. We conducted systematic retrieval of prospective studies in PubMed, Embase, and Web of Science. Summary relative risks were compiled with a fixed effects model or a random effects model, and a restricted cubic spline regression model and generalized least squares analysis were used to evaluate the diet–T2D incidence relationship. T2D risk increased with increasing consumption of total protein and animal protein, red meat, processed meat, milk, and eggs, respectively, while plant protein and yogurt had an inverse relationship. A non-linear association with the risk for T2D was found for the consumption of plant protein, processed meat, milk, yogurt, and soy. This meta-analysis suggests that substitution of plant protein and yogurt for animal protein, especially red meat and processed meat, can reduce the risk for T2D.

## 1. Introduction

Diabetes mellitus is considered a serious public health issue worldwide with the vast majority of patients having type 2 diabetes (T2D). According to the International Diabetes Federation, T2D is expected to grow by 54.5 percent from 2013 levels by 2035 [1]. Accordingly, T2D is a tremendous burden on the economic and social medical security system [2].

Dietary factors are major behavioral factors that can influence the risk for T2D to a great extent [3]. One study found that the development of T2D is particularly sensitive to dietary factors, in comparison to other chronic diseases, like cerebrovascular disease or cancer [4].The relationship between protein consumption and T2D incidence has been inconsistently presented. A meta-analysis of this topic pointed out total protein intake may increase the risk of T2D [5], while long-term follow-up observational studies have suggested that high protein intake is not an independent risk factor for T2D [6]. Several longitudinal studies have observed that animal protein including red and processed meat is positively associated with T2D risk [7,8,9]; however, plant-based food which have high protein content is associated with a lower risk of T2D [10,11].

There are a growing number of studies investigating the potential link between protein intake and diabetes, and the conclusions are not consistent. The relationship between protein consumption and high-protein food intake with T2D risk has not been systematically evaluated. To address this question, we performed a meta-analysis of prospective studies to quantify this relationship and identify optimal food types for a low T2D risk.

## 2. Methods

### 2.1. Literature Search and Study Design

We identified cohort studies that looked at the relationship between dietary protein intake and T2D by searching the databases of PubMed, Embase, and Web of Science as of 5 March 2019. The full search strategy is presented in Supplementary Table S1; Studies were limited to those in human subjects. If the same cohort has been studied multiple times in different literatures, the latest data were used. In addition, references for the included studies were manually retrieved.

### 2.2. Study Selection

All included studies matched the following criteria: the study must have a cohort design; the study outcomes include type 2 diabetes; the study had to report relative ratio (RR), odds ratio (OR), or hazard ratio (HR); the independent variables for the study included protein intake; and all studies had a quality score as assessed by the Newcastle Ottawa Scale (NOS) of cohort studies of at least 6.

### 2.3. Data Extraction and Quality Assessment

Two authors independently extracted the following information from each publication: the first author, publication year, geographic location, study name, cohort size, the years of follow-up, gender, baseline age of study subjects, diagnosis and assessment of T2D, dietary assessment methods, type of dietary protein, number of participants and T2D cases, dietary protein consumption per category and the RR/OR/HR and 95% CI of T2D incidence related to those categories, number of T2D cases and total subjects per category, and covariates of a fully adjusted model included in the analyses. The dispute was resolved by discussion between the two authors.

Two reviewers independently performed the quality assessment by using the NOS (for cohort and case-control studies) with a full score of 9 stars [12].

### 2.4. Statistical Methods

If the category of dietary protein intake had an open interval, we assumed that the width of the interval is the same as the adjacent category [13]. For cohort studies reporting HRs or ORs for T2D, we assumed that the HR was RR and transformed the OR to RR by a formula as previously described [14]. 

We initially pooled RRs and the corresponding 95% CIs from the highest compared with the lowest category of dietary protein intake in each study by the fixed-effect or random-effect model [15]. To capture the detailed nature of the association, the method of generalized least squares regression [16] was used to estimate the linear dose-response relationship for each increment of 5% energy from protein intake, 50 g of meat, fish, poultry, legumes and egg intake (one egg/day), 100 g of milk and yogurt intake, and 30 g of cheese intake. We explored potential non-linear associations by two-stage restricted cubic spline analysis, with three knots at the 25th, 50th, and 75th percentiles of the protein intake distribution [14]. A *p* value for non-linearity was calculated by testing whether the coefficient of the second spline was equal to zero [17]. Subgroup analyses were conducted by gender (men, women, men and women), age (<50 vs. ≥50 years), follow-up time (<10 vs. ≥10 years), geographic location (Europe, America, Asia, and Australia), and number of cases (<1000 vs. ≥1000).

Heterogeneity among studies was assessed using the Q test and I^2^ statistic (If the value of I^2^ > 50%, then heterogeneity may exist) [18]. We assessed publication bias by Begg-Mazumdar’s test and Egger’s tests [19]. A two-sided *p* value was used with a level of 0.05. All data analyses were performed by using Stata 12.1 (Stata Corp., College Station, TX, USA).

## 3. Results

### 3.1. Literature Search and Included Study Characteristics

**Figure 1:**
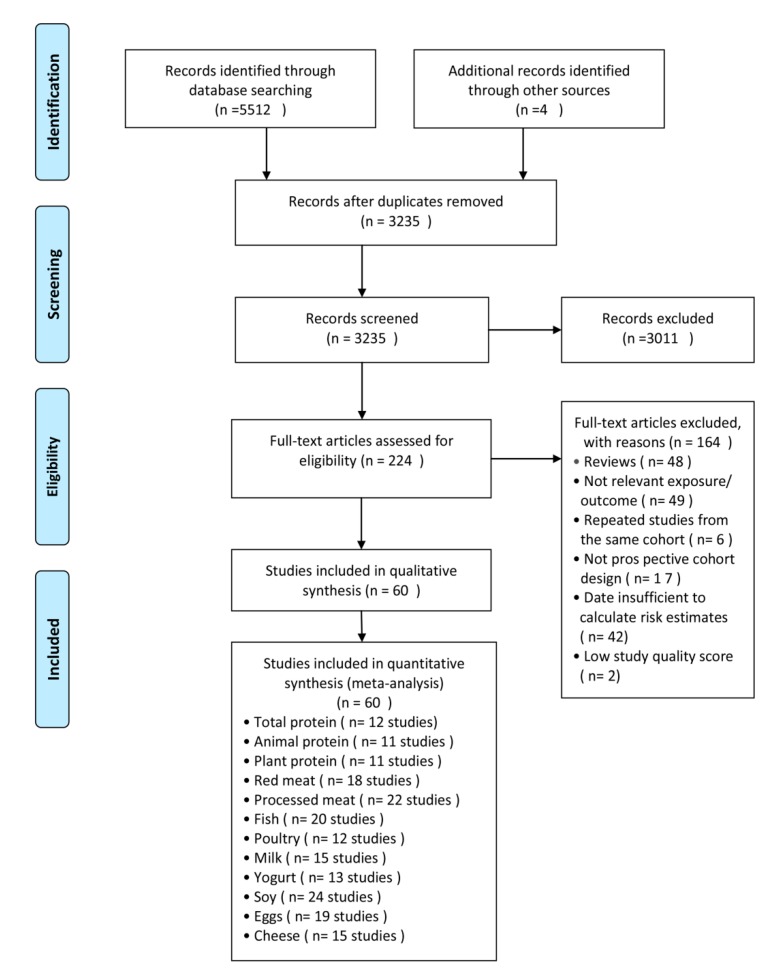
Flowchart for study selection.

A flowchart (Figure 1) shows the process of research selection. In short, we identified a total of 1919 records in PubMed, 2461 in Embase, 1132 in Web of Science and four from searched references, of which 5292 records were excluded after duplicates were removed and a review of title and abstract; 164 records were further deleted due to violations of the required standards. Finally, 60 articles with 72 studies including 2,623,984 subjects and 141,471 T2D were analyzed in this meta-analysis. Supplementary Tables S2–S6 reveals the characteristics of the 72 studies. And of the included studies, 27 were conducted in USA, 23 in Europe, 17 in Asia, three in Finland, and two in Australia. As displayed in Supplementary Table S7, all the studies were of high quality with scores above six.

### 3.2. Dietary Protein Intake and Risk of T2D

#### 3.2.1. Total Protein

**Figure 2:**
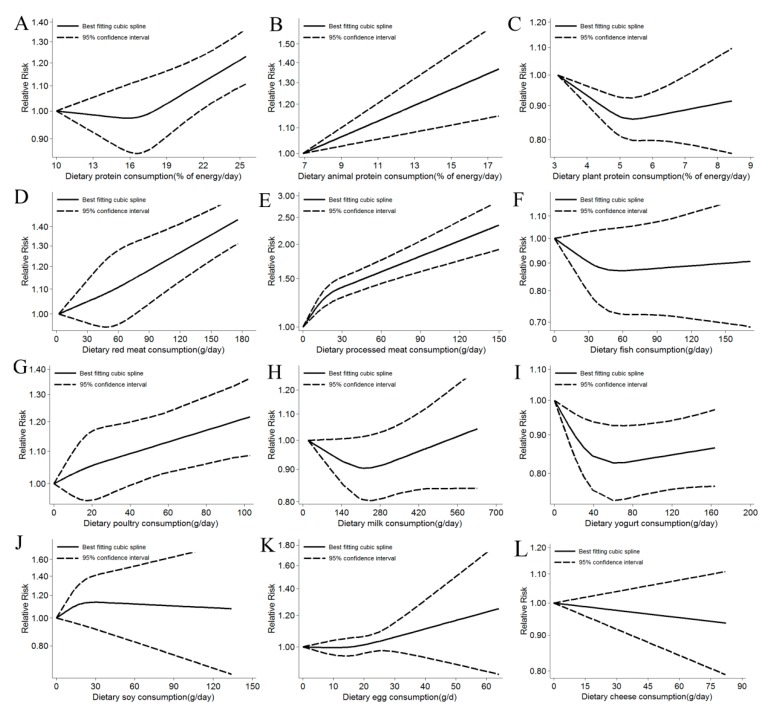
Non-linear dose-response relationship between daily intake of total protein (**A**) (*p* non-linearity = 0.084), animal protein (**B**) (*p* non-linearity = 0.780), plant protein (**C**) (*p* non-linearity = 0.003), red meat (**D**) (*p* non-linearity = 0.777), processed meat (**E**) (*p* non-linearity < 0.001), fish (**F**) (*p* non-linearity = 0.119), poultry (**G**) (*p* non-linearity = 0.929), milk (**H**) (*p* non-linearity = 0.042), yogurt (**I**) (*p* non-linearity = 0.004), soy (**J**) (*p* non-linearity = 0.028), eggs (**K**) (*p* non-linearity = 0.402), and cheese (**L**) (*p* non-linearity = 0.216) and risk of T2D.

Twelve studies [5,6,20,21,22,23] were included in the dose-response meta-analysis which showed a significantly positive association for protein intake with T2D incidence. For a 5% of energy increment from total protein, the RR of T2D was 1.08 (95% CI: 1.05, 1.11, I^2^ = 0.0%) (Supplementary Figure S1). The positive associations persisted in the high vs. low intake meta-analysis (RR: 1.11; 95% CI: 1.05, 1.16, I^2^ = 5.1%, *n* = 12 studies) and there was no heterogeneity in stratified analyses (Supplementary Table S8).There was little evidence of a non-linear association (*p* = 0.084) (Figure 2).

#### 3.2.2. Animal Protein

Eleven studies [5,6,20,22,23,24] showed that animal protein intake increased the risk of T2Din the dose-response meta-analysis. For a 5% of energy increment from animal protein, the RR of T2D was 1.11 (95% CI: 1.07, 1.15, I^2^ = 42.7%) (Supplementary Figure S2). The result of the high vs. low intake meta-analysis was similar to the per 5% increase (RR: 1.13; 95% CI: 1.08, 1.19, I^2^ = 14.3%, *n* = 11 studies) and we detected evidence of heterogeneity in analysis of gender, countries, and number of cases (Supplementary Table S9). There was no evidence of a non-linear association (Figure 2).

#### 3.2.3. Plant Protein

An inverse association was observed with plant protein in 10 studies [5,6,20,22,23,24] included in the dose-response meta-analysis. For a 5% of energy increment from plant protein, the RR of T2D was 0.85 (95% CI: 0.76, 0.96, I^2^ = 41.7%) (Supplementary Figure S3). A consistent result was also observed for the high versus low intake relationship (RR: 0.93; 95% CI: 0.87, 0.99, I^2^ = 0.0%, *n* = 11 studies) and there was no evidence of heterogeneity between subgroups (Supplementary Table S10). In non-linear dose-response association analysis, we observed that the risk of T2D was decreased by 14% with increasing intakes of plant protein up to 5% of total daily energy (Figure 2).

#### 3.2.4. High-Protein Food Intake and the Risk of T2D

##### Red Meat

In the dose-response meta-analysis, each 50 g of red meat intake per day increased the risk of T2D (RR: 1.11; 95% CI: 1.06, 1.16, I^2^ = 76.0%, *n* = 16 studies) (Supplementary Figure S4). The positive associations and heterogeneity persisted in the high vs. low intake meta-analysis (RR: 1.22; 95% CI: 1.14, 1.29, I^2^ = 60.3%, *n* = 18 studies [6,7,21,24,25,26,27,28,29,30,31,32,33]) and stratified analysis (Supplementary Table S11).There was no evidence of a non-linear association (Figure 2).

##### Processed Meat

Sixteen studies were included in the dose-response meta-analysis. Each additional daily consumption of 50 g of processed meat was strongly associated with T2D risk (RR: 1.41; 95% CI: 1.24, 1.60, I^2^ = 85.6%) (Supplementary Figure S5). The similar result was also detected in the high vs. low intake meta-analysis (RR: 1.25; 95% CI: 1.15, 1.35, I^2^ = 77.3%, *n* = 22 studies [6,7,21,24,26,27,28,29,30,31,32,33,34,35,36,37,38]), and heterogeneity was found in both the master study and the stratified analysis (Supplementary Table S12). The evidence of non-linear analysis showed that the risk of T2D doubled with increasing intake of processed meat up to 150 g/day compared with no processed meat intake (Figure 2).

##### Fish

No association was observed in the dose-response meta-analysis of all participants in eight studies for each daily 50g of fish intake (RR: 0.99; 95% CI: 0.92, 1.07, I^2^ = 61.9%) (Supplementary Figure S6). A borderline positive association was observed in the high vs. low intake meta-analysis (RR: 1.08; 95% CI: 1.00, 1.18, I^2^ = 76.6%, *n* = 20 studies [6,20,21,25,26,28,39,40,41,42,43,44,45,46,47]) (Supplementary Table S13). Moreover, an inverse association was found for studies in men and in Asia. There was no evidence of a non-linear association (Figure 2).

##### Poultry

Eight studies included in this analysis showed no linear dose-response association between poultry consumption and T2D risk (RR: 1.02; 95% CI: 0.98, 1.07, I^2^ = 0.0%) (Supplementary Figure S7). A slight inverse association was detected when comparing the highest intake with the lowest intake category (RR: 1.04; 95% CI: 1.00, 1.08, I^2^ = 17.7%, *n* = 12 studies [25,26,27,28,29,30,31,32,33,48]) and no heterogeneity was found in either the master analysis or the stratified analysis (Supplementary Table S14). No evidence of a non-linear association was detected (Figure 2).

##### Milk

In the dose-response meta-analysis, a slight positive association was observed for each daily 100g of milk consumed in seven studies (RR: 1.01; 95% CI: 1.00, 1.03, I^2^ = 5.3%) (Supplementary Figure S8). Comparing categories of highest and lowest intake of milk, we observed no association with the risk of T2D (RR: 0.98; 95% CI: 0.93, 1.02, I^2^ = 27.5%, *n* = 15 studies [6,33,40,47,48,49,50,51,52,53,54,55,56,57]) (Supplementary Table S15). However, in the non-linear dose-response association analysis, we observed that eating 200 g of milk a day reduced the risk of T2D by 9% (Figure 2).

##### Yogurt

Nine studies were included in the dose-response meta-analysis and a negative association was observed for each daily 100g of yogurt consumption (RR: 0.86; 95% CI: 0.81, 0.92, I^2^ = 48.9%) (Supplementary Figure S9). A significant inverse association was also observed for the highest versus lowest yogurt intake categories (RR: 0.83; 95% CI: 0.77, 0.89, I^2^ = 29.4%, *n* = 12 studies [40,49,50,52,54,55,56,58,59]). The inverse association and heterogeneity remained in hierarchical analysis except for gender (Supplementary Table S16). In addition, the risk of T2D decreased by 17% with increasing intake of yogurt up to 60 g/day in non-linear analysis (Figure 2).

##### Soy

No association was observed in the dose-response meta-analysis with regard to soy protein intake (RR: 1.15; 95% CI: 0.97, 1.37, I^2^ = 85.2%, *n* = 19 studies) (Supplementary Figure S10). A similar result was found for the highest versus lowest soy intake category (RR: 1.00; 95% CI: 0.90, 1.10, I^2^ = 82.0%, *n* = 24 studies [11,20,21,28,57,60,61,62,63,64,65]) and evidence of heterogeneity between subgroups was observed for gender and number of cases (Supplementary Table S17). The association was not statistically significant in non-linear dose-response association (Figure 2).

##### Eggs

Nineteen studies were included in the dose-response meta-analysis and a borderline positive association was observed for each one egg consumption per day (RR: 1.01; 95% CI: 0.99, 1.03, I^2^ = 70.8%) (Supplementary Figure S11). A significant positive association of the highest vs. lowest egg intake category was detected (RR: 1.10; 95% CI: 1.03, 1.16, I^2^ = 54.8%, *n* = 19 studies [6,28,33,47,48,66,67,68,69,70,71,72,73,74]) (Supplementary Table S18). The heterogeneity still exited in stratified analyses and there was no evidence of a non-linear dose–response association (Figure 2).

##### Cheese

Twelve studies were included in the dose-response meta-analysis and no association was observed for each daily 30g of cheese consumption (RR: 0.97; 95% CI: 0.93, 1.02, I2 = 13.9%) (Supplementary Figure S12). A borderline negative association of the highest versus lowest cheese intake category was detected (RR: 0.94; 95% CI: 0.89, 1.00, I^2^ = 1.9%, *n* = 15 studies [6,47,48,49,50,51,52,54,55,56,58,59]) and there was no evidence of heterogeneity between subgroups in stratified analyses (Supplementary Table S19). Moreover, a significant inverse association was observed for studies in women and in USA. No evidence of a non-linear association was detected (Figure 2).

### 3.3. Summary across Food Groups

Table 1 shows the RR for T2D from the non-linear dose-response analysis of the nine food groups according to daily intake. Optimal intake of risk-decreasing food (60 g/day of yogurt and 200 g/day of milk) results in a 24% reduction ($1-{\mathrm{RR}}_{\mathrm{reduced}}^{*}$) compared to non-consumption. Risk-increasing foods were red meat and processed meat. Compared with non-consumption, consumption of two servings red meat daily (170 g, RR: 1.36) and three serving processed meat daily (150 g, RR: 2.34) was associated with about a three-fold increased risk (${\mathrm{RR}}_{\mathrm{increased}}^{*}$). In addition, if these foods not eat, the risk of T2Dwould be reduced by 69% (1 − $\frac{1}{{\mathrm{RR}}_{\mathrm{increased}}^{*}}$).

### 3.4. Risk of Bias

The results varied little by methodological assumption, including only those with a low risk of bias (Supplementary Tables S8–S19).

### 3.5. Publication Bias

In this meta-analysis, no publication bias was found by Begg-Mazumdar’s test and Egger’s test (Supplementary Figures S13–S24), except for the fish consumption analysis (Table 2); visual inspection of the funnel plot suggested that there was evidence for small study effects of fish consumption and risk of T2D (Supplementary Figure S18).

## 4. Discussion

A comprehensive meta-analysis of 72 studies demonstrated that consumption of total and animal protein was positively associated with the risk for T2D and intake of plant protein was inversely related to the risk of T2D. In the linear dose-response meta-analysis of nine food groups and their relationship to T2D incidence, the intake of red meat, processed meat, milk, and eggs, the relationship was positive with T2D incidence; only the association between yogurt consumption and T2D yielded an inverse relationship. Some evidence of non-linearity between the consumption of plant protein, processed meat, milk, yogurt, and soy with the risk of T2D was found.

There is consistent evidence that total and animal protein increases the risk of T2D, and a beneficial effects of plant protein consumption on diabetes is in line with a previous meta-analysis [5,75,76]. A meta-analysis of randomized controlled trials showed that substituting plant protein for animal protein was beneficial for glycemic control [77]. The mechanism of action of protein types on T2D seems to be undefined and equivocal. Our study supports the hypothesis that protein quality plays a more vital role than protein quantity in T2D risk [6]. The type and abundance of amino acids differ between animal and plant proteins and may contribute to the differing risk associated with these proteins. It has been noted that animal protein contains higher levels of the branched chain amino acids compared with plant proteins, and leucine, isoleucine, and methionine may be risk factors for insulin resistance and T2D [78]. Glycine, an amino acid that was found to be associated with a higher risk of T2D, is also abundant in animal protein [79,80].

Although excessive consumption of total animal protein is likely to augment the risk of T2D, not all foods containing high animal protein are equal. A positive association was found for meat intake (especially processed meat) and egg consumption. On the contrary, white meat and dairy products may be beneficial for T2D prevention. Thus, the complex effect of animal protein on diabetes is remains to be established.

The results for red and processed meat were aligned with an updated meta-analysis which showed a 19% and 51% higher risk of T2D for 100 g red meat/day and 50 g processed meat/day, respectively [7]. A study of Caucasians revealed that processed meat was related to higher fasting glucose, and red meat was related to both higher fasting glucose and fasting insulin concentrations [81]. Moreover, saturated fat may contribute to obesity, a major risk factor for glucose intolerance, insulin resistance and diabetes, and red meat and processed meat are high in this content, so these meat intake may lead to the formation of a causal pathway of T2D [82]. The potential mechanisms by which diets with different meat composition influence T2D risk require further investigation.

Although no association was detected in the meta-analysis of fish consumption and risk of T2D, stratification analysis showed that fish consumption was associated with a lower risk of T2D in Asian studies but not in Europe. It has been reported that fish consumption is negatively associated with the risk of T2D in China [39], while a case-cohort study from eight European countries found no association [83]. To the best of our knowledge, our study is the first meta-analysis to identify an association of poultry intake with the risk of T2D. The subgroup analysis and a recent study both showed a protective effect of poultry intake on the development of T2D in China [84]. Regional differences in the relationship between white meat intake and diabetes may be related to the distinct in cooking styles between China and Europe, particularly in regard to frying versus boiled or steamed meat. Cooking at high temperatures, as with frying, may lead to the formation of glycation end products, which may cause insulin resistance, and frying may further contribute to a loss of long-chain omega-3 fatty acids and induce the formation of mutagenic compounds, such as heterocyclic amines [41].

In this study, intake of yogurt was inversely with the risk of T2D at any daily intake. The risk of T2D was reduced by 17% when the consumption of yogurt was up to 60 g/day. The study was reinforced by a previous review that suggested dairy products, especially fermented yogurt, may reduce the risk of T2D by reducing the risk of obesity, one of the main causes of T2D [85]. The favorable effect may be attributed to the calcium, potassium, and vitamin D in dairy products [86]. A randomized, double-blind, placebo-controlled study suggested that probiotic (as in yogurt) consumption improved glycemic control in T2D subjects [87]. An inverse association of consumption of cheese and risk of T2D was presented for studies in the United States and women. The results align with a previous meta-analysis and a recent narrative review, which both found that the benefit effect of dairy was largely contributed to low-fat dairy, especially yogurt and cheese [88,89]. However, milk consumption was associated with a weakly reduced risk of T2D only in the non-linear dose-response meta-analysis, which is inconsistent with previous studies that have suggested that milk intake is not clearly associated with T2D [90,91]. As a result of these inconsistencies, further research will be needed to offer convincing evidence.

This study suggests a slight detrimental effect of egg consumption for the risk of T2D, which was supported by the American Heart Association (AHA) dietary guidelines that egg intake should be limited to prevent cardiovascular disease [92]. Some evidence has suggested that intestinal flora affects the production of trimethylamine-*N*-oxide from egg yolks which might be a driver for the positive association between eggs and T2D [93]. Contradictory to our findings, a previous meta-analysis showed that fasting blood glucose was not affected by moderate egg intake (3 eggs/week) [68]. Moreover, eggs are widely consumed worldwide and rich in high quality protein with low saturated fat, but the high cholesterol and choline levels raise health concerns [94].Therefore, it is necessary to study further the relationship between egg intake and glucose metabolism across populations.

For high protein plant-based foods, no association was detected for soy consumption and the risk of T2D in all subjects. However, it was found that soy consumption could reduce the risk of T2D in Asians but not in Europeans and Americans. And, a study from Thailand, an Asian country, and a cross-sectional study from China both observed that traditional fermented soy intake had a slight protective effect on glucose [95,96]. Such a distinction could be due to the fact that there is a higher average intake of soy in Asians than in Europeans and Americans. Several beneficial components of soy products may contribute to the hypoglycemic effect, including isoflavones and soy protein [97]. A cell-based study suggested that soy protein and isoflavones are beneficial for reducing the risk of onset and development of insulin resistance and T2D and a review also showed that fermented soy products may attenuate the progression of T2D by improving insulin resistance and insulin secretion [98,99]. A pooled analysis reported that isoflavones can improve hyperglycemia, glucose tolerance, and circulating insulin concentration, and can also stimulate phosphorylated AMP-activated protein kinase and acetyl-CoA carboxylase to increase glucose uptake and fatty acid oxidation [60].

A comprehensive comparison of dietary guidelines around the world shows that different countries have similar dietary recommendations for some daily food, such as strict limits on the consumption of processed meat, whether among healthy people or patients with chronic diseases. Our meta-analysis provides further scientific evidence to support the inclusion of some food groups in the food-based dietary guidelines and to understand their potential role in the risk for T2D. Based on our study, the optimal consumption of yogurt and milk is associated with 24% lower risk, whereas avoidance of foods associated with a higher risk is associated with 69% lower incidence of T2D. Establishing a proper amount of total protein intake, strictly limiting the intake of animal protein, especially for processed meat and red meat, and maintaining adequate intake of yogurt and plant protein, can produce a host of beneficial effects to reduce the risk of T2D in a convenient and cost-effective way.

Foremost in the strengths of this meta-analysis is the large number and high quality of included prospective studies and a complete and detailed description of dietary protein consumption. Another strength is the variety of types of analyses including a high vs. low meta-analysis, linear dose–response analysis, and non-linear dose-response analysis. Third, we effectively avoided recall bias because the studies we included were cohort studies.

Some limitations also exist in this meta-analysis. First, some studies did not adjust for important factors that influence T2D such as family history of diabetes and physical activities, which could compromise results. Second, among the included studies, the follow-up time span was large, so a temporal bias may affect the relationship between dietary protein consumption and T2D risk. Third, some food groups showed heterogeneity with respect to the analyzed age, sex, follow-up length, geographic location, and number of cases.

## 5. Conclusions

Among the investigated protein and high-protein foods, selecting specific optimal intakes (by increasing plant protein and yogurt; and reducing total protein, animal protein including red and processed meat, and eggs) can lead to a significant decrease in risk for T2D.

## Figures and Tables

**Table 1:** RRs from the non-linear dose-response analysis of nine pre-defined food groups and the risk of T2D according to intake of servings per day.

Servings/d	Food Group and Daily Serving Size		
	Inverse association		
	Yogurt (1 serving = 30 g)	Milk (1 serving = 100 g)	
Ref.	1.00	1.00	
1	0.87 (0.79, 0.95)	0.94 (0.88, 1.00)	
2	0.83 (0.74, 0.93)	0.91 (0.81, 1.00)	
3	0.83 (0.75, 0.93)	0.92 (0.81, 1.03)	
4	0.85 (0.76, 0.94)	0.95 (0.83, 1.09)	
5	0.86 (0.77, 0.96)	0.97 (0.82, 1.09)	
6	NA	NA	
	Positive association		
	Red meat (1 serving = 85 g)	Processed meat (1 serving = 50 g)	
Ref.	1.00	1.00	
1	1.17 (1.02, 1.34)	1.51 (1.38, 1.65)	
2	1.36 (1.22, 1.52)	1.81 (1.57, 2.03)	
3	NA	2.34 (1.91, 2.87)	
4		NA	
	No association		
	Poultry (1 serving = 50 g)	Fish (1 serving = 50 g)	Egg (1 serving = 1 egg)
Ref.	1.00	1.00	1.00
1	1.10 (0.99, 1.22)	0.87 (0.73, 1.04)	1.16 (0.90, 1.50)
2	NA	0.88 (0.72, 1.09)	NA
3		0.90 (0.69, 1.18)	
4		NA	
	Soy (1 serving = 50 g)		Cheese (1 serving = 30 g)
Ref.	1.00		1.00
1	1.14 (0.87, 1.50)		0.92 (0.83, 1.01)
2	1.11 (0.73, 1.71)		0.91 (0.81, 1.02)
3	NA		NA

Ref.: no consumption of nine pre-defined food groups.

**Table 2:** Publication bias and meta-analysis.

Proteins and Foods Sources	Begg-Mazumdar’s Test	Egger’s Test
Total protein	0.304	0.192
Animal protein	0.161	0.102
Plant protein	0.107	0.110
Red meat	0.964	0.720
Processed meat	0.192	0.208
Fish	0.035	0.011
Poultry	0.902	0.943
Milk	0.548	0.313
Yogurt	0.466	0.357
Soy	0.184	0.076
Egg	0.108	0.021
Cheese	0.755	0.656

## Supplementary Materials

The following are available online at https://www.mdpi.com/2072-6643/11/11/2783/s1. Table S1: Search terms in meta-analysis of association between dietary protein and type 2 diabetes, Table S2: Characteristics of included studies of protein consumption and type 2 diabetes, Table S3: Characteristics of included studies of meat consumption and type 2 diabetes, Table S4: Characteristics of included studies of total dairy consumption and type 2 diabetes, Table S5: Characteristics of included studies of soy consumption and type 2 diabetes, Table S6: Characteristics of included studies of egg consumption and type 2 diabetes, Table S7: Quality assessment of cohort studies included in meta-analysis(Newcastle-Ottawa Quality Assessment Scale),Table S8: Dose-response meta-analysis for per 5% of energy/day increase in total protein and risk of type 2 diabetes, stratified by high vs. low intake, gender, age, follow-up, geographic location, number of cases, Table S9: Dose-response meta-analysis for per 5% of energy/day increase in animal protein and risk of type 2 diabetes, stratified by high vs. low intake, gender, age, follow-up, geographic location, number of cases, Table S10: Dose-response meta-analysis for per 5% of energy/day increase in plant protein and risk of type 2 diabetes, stratified by high vs. low intake, gender, age, follow-up, geographic location, number of cases, Table S11: Dose-response meta-analysis for each 50 g/day increase in red meat and risk of type 2 diabetes, stratified by high vs. low intake, gender, age, follow-up, geographic location, number of cases, Table S12: Dose-response meta-analysis for each 50 g/day increase in processed meat and risk of type 2 diabetes, stratified by high vs. low intake, gender, age, follow-up, geographic location, number of cases, Table S13: Dose-response meta-analysis for each 50 g/day increase in fish and risk of type 2 diabetes, stratified by high vs. low intake, gender, age, follow-up, geographic location, number of cases, Table S14: Dose-response meta-analysis for each 50 g/day increase in poultry and risk of type 2 diabetes, stratified by high vs. low intake, gender, age, follow-up, geographic location, number of cases, Table S15: Dose-response meta-analysis for each 100 g/day increase in milk and risk of type 2 diabetes, stratified by high vs. low intake, gender, age, follow-up, geographic location, number of cases, Table S16: Dose-response meta-analysis for each 100 g/day increase in yogurt and risk of type 2 diabetes, stratified by high vs. low intake, gender, age, follow-up, geographic location, Table S17: Dose-response meta-analysis for each 50 g/day increase in soy and risk of type 2 diabetes, stratified by high vs. low intake, gender, age, follow-up, geographic location, number of cases, Table S18: Dose-response meta-analysis for each 50 g/day(one/day) increase in egg and risk of type 2 diabetes, stratified by high vs. low intake, gender, age, follow-up, geographic location, number of cases, Table S19: Dose-response meta-analysis for each 30 g/day increase in cheese and risk of type 2 diabetes, stratified by high vs. low intake, gender, age, follow-up, geographic location, number of cases, Figure S1: Prospective associations of dietary total protein intake with incident type 2 diabetes for per 5% of energy increase, Figure S2: Prospective associations of dietary animal protein intake with incident type 2 diabetes for per 5% of energy increase, Figure S3: Prospective associations of dietary plant protein intake with incident type 2 diabetes for per 5% of energy increase, Figure S4: Prospective associations of dietary red meat intake with incident type 2 diabetes for per 50 g/day increase, Figure S5: Prospective associations of dietary processed meat intake with incident type 2 diabetes for per 50 g/day increase, Figure S6: Prospective associations of dietary fish intake with incident type 2 diabetes for per 50 g/day increase, Figure S7:Prospective associations of dietary poultry intake with incident type 2 diabetes for per 50 g/day increase, Figure S8: Prospective associations of dietary milk intake with incident type 2 diabetes for per 100 g/day increase, Figure S9: Prospective associations of dietary yogurt intake with incident type 2 diabetes for per 100 g/day increase, Figure S10: Prospective associations of dietary soy intake with incident type 2 diabetes for per 50 g/day increase, Figure S11: Prospective associations of dietary egg with incident type 2 diabetes for per one egg/day increase, Figure S12: Prospective associations of dietary cheese with incident type 2 diabetes for per 30 g/day increase, Figure S13: Funnel plots of dietary total protein-T2Dassociations (dose-responsemeta-analysis) SE = Standard error, Figure S14: Funnel plots of dietary animal protein-T2Dassociations (dose-response meta-analysis) SE = Standard error, Figure S15: Funnel plots of dietary plant protein-T2Dassociations (dose-response meta-analysis) SE = Standard error, Figure S16: Funnel plots of dietary red meat-T2Dassociations (dose-response meta-analysis) SE = Standard error, Figure S17: Funnel plots of dietary processed meat-T2Dassociations (dose-response meta-analysis) SE = Standard error, Figure S18: Funnel plots of dietary fish-T2Dassociations (dose-response meta-analysis) SE = Standard error, Figure S19: Funnel plots of dietary poultry-T2Dassociations (dose-response meta-analysis) SE = Standard error, Figure S20: Funnel plots of dietary milk-T2Dassociations (dose-response meta-analysis) SE = Standard error, Figure S21: Funnel plots of dietary yogurt-T2Dassociations (dose-response meta-analysis) SE = Standard error, Figure S22: Funnel plots of dietary soy-T2Dassociations (dose-response meta-analysis) SE = Standard error, Figure S23: Funnel plots of dietary egg-T2Dassociations (dose-response meta-analysis) SE = Standard error, Figure S24: Funnel plots of dietary cheese-T2D associations (dose-response meta-analysis) SE = Standard error.
