# Supplementary material for: Dietary Protein Consumption and the Risk of Type 2 Diabetes: ADose-Response Meta-Analysis of Prospective Studies
Source: Nutrients. 2019 Nov 15;11(11):2783. doi: 10.3390/nu11112783 (PMC6893550; doi:10.3390/nu11112783)
Supplement: Supplementary file 1 [file nutrients-11-02783-s001.pdf]

Supplementary tables:

Supplementary table 1 Search terms in meta-analysis of association between dietary protein and type 2 diabetes

Search theme	Search date	Pubmed search term	Embase search term
Protein	2019.3.5	("Dietary Proteins"[Mesh] OR "dietary protein"[tiab]OR "dietary proteins"[tiab] OR "proteinconsumption"[tiab] OR "protein intake"[tiab] OR "animalprotein"[tiab]OR "plant protein"[tiab] OR "vegetable protein" [tiab]) AND ("DiabetesMellitus"[Mesh] OR "diabetes" [tiab]) AND ("Cohort Studies"[Mesh] OR "prospective"[tiab] OR "longitudinal"[tiab] OR "cohort"[tiab])	("dietary protein" OR "dietary proteins" OR "protein consumption" OR "protein intake"OR "animal protein" OR "plant protein" OR "vegetable protein") AND ("diabetes mellitus") AND ("prospective" OR "longitudinal" OR "cohort")
Meat	2019.3.5	("Meat"[Mesh] OR"meat"[tiab] OR"red meat"[tiab] OR"processed meat"[tiab]OR"totalmeat"[tiab]) AND ("DiabetesMellitus"[Mesh] OR "diabetes" [tiab]) AND ("Cohort Studies"[Mesh] OR "prospective"[tiab] OR "longitudinal"[tiab] OR "cohort"[tiab])	("meat" OR "red meat" OR "processed meat"OR"totalmeat") AND ("diabetes mellitus") AND ("prospective" OR "longitudinal" OR "cohort")
Fish	2019.3.5	("Fishes"[Mesh]OR "fishes"[tiab]OR "fish protein"[tiab] OR "seafood"[tiab]OR "fish"[tiab]) AND ("DiabetesMellitus"[Mesh] OR "diabetes" [tiab]) AND ("Cohort Studies"[Mesh]OR "prospective"[tiab] OR "longitudinal"[tiab] OR "cohort"[tiab])	("fishes"OR"fish protein" OR"seafood" OR "fish") AND ("diabetes mellitus") AND ("prospective" OR "longitudinal" OR "cohort")
Poultry	2019.3.5	("Poultry"[Mesh]OR "poultry "[tiab]OR " poultry protein"[tiab] OR "poultry products"[tiab]) AND ("DiabetesMellitus"[Mesh] OR "diabetes" [tiab]) AND ("Cohort Studies"[Mesh]OR "prospective"[tiab] OR "longitudinal"[tiab] OR "cohort"[tiab])	("poultry "OR" poultry protein" OR" poultry products ") AND ("diabetes mellitus") AND ("prospective" OR "longitudinal" OR "cohort")
Egg	2019.3.5	("Eggs"[Mesh]"eggs"[tiab]OR "egg"[tiab] OR "egg protein"[tiab]) AND ("Diabetes Mellitus"[Mesh]OR "diabetes" [tiab]) AND ("Cohort Studies"[Mesh]OR "prospective"[tiab] OR "longitudinal"[tiab] OR "cohort"[tiab])	("egg" OR "eggs"OR "egg protein") AND ("diabetes mellitus") AND ("prospective" OR "longitudinal" OR "cohort")
Diary	2019.3.5	("Milk"[Mesh]OR "milk"[tiab]OR "dairy"[tiab] OR "dairy product"[tiab]OR "cheese"[tiab] OR "butter"[tiab]OR "cream"[tiab]OR "yogurt"[tiab]) AND ("Diabetes Mellitus"[Mesh] OR "diabetes"[tiab]) AND ("Cohort Studies"[Mesh] OR "prospective"[tiab] OR "longitudinal"[tiab] OR "cohort"[tiab])	("milk" OR "dairy" OR "dairy product" OR "cheese"OR "butter"OR "cream" OR "yogurt") AND ("diabetes mellitus") AND ("prospective"OR "longitudinal" OR "cohort")
Soy	2019.3.5	("Soybeans"[Mesh]OR "Soybeans"[tiab]OR "soy"[tiab] OR "legume"[tiab] OR "soy product" [tiab] OR "soy isoflavones" [tiab]) AND ("Diabetes Mellitus"[Mesh] OR "diabetes" [tiab]) AND ("Cohort Studies"[Mesh] OR "prospective"[tiab] OR "longitudinal"[tiab] OR "cohort"[tiab])	("Soybeans" OR "soy" OR "legume" OR "soy product"OR "soy isoflavones") AND ("diabetes mellitus") AND ("prospective" OR "longitudinal" OR "cohort")
Pubmed	2019.3.5	Protein+ Meat +Fish +Poultry+Egg +Diary +Soy	
Embase	2019.3.5	Protein+ Meat +Fish +Poultry+Egg +Diary +Soy	
Web of Science	2019.3.5	Topic="dietary protein" OR "dietary proteins" OR"protein consumption" OR "protein intake" OR "animal protein" OR "plant protein" OR "vegetable protein" OR "meat" OR"red meat" OR"processed meat"OR"fishes"OR "fish protein"OR "seafood"OR "fish"OR "poultry"OR "poultry products"OR "egg"OR "egg protein"OR "milk"OR "dairy" OR "dairy product"OR"yogurt"OR "Soybeans"OR "soy"OR "legume"AND Topic="diabetes" AND Topic="prospective" OR "longitudinal" OR "cohort"	

Supplementary table2 Characteristics of included studies of protein consumption and type 2 diabetes

First Author	Publication year	Study name	Country	Follow-up years	Population	Method of dietary exposure assessment and type of protein	Method of diabetes assessment	Adjustment variables
Chen	2019	RS-I<br>RS-II<br>RS-III	Netherlands	7.2	≥45;<br>41.4%men;<br>643 cases,<br>6813 subjects	170-item FFQ,<br>389-item FFQ;<br>Total protein,<br>Animal protein,<br>Plant protein	1.Fasting blood glucose concentration of 7.0mmol/L or higher<br>2.Non- fasting blood glucose concentration of 11.1mmol/L or higher<br>3. The use of hypo-glycemic drugs	Total fat intake, total energy intake, alcohol intake, age, sex,smoking status,education level, diet quality score,physical activity and family history of diabetes, time point of WC measurements, longitudinal WC, the interaction between protein intake andlongitudinal WC
Virtanen	2017	KIHD	Finland	19.3	53.1±5.2;<br>100%men;<br>432 cases,<br>44933 subjects	4-d dietary record;<br>Total protein,<br>Animal protein,<br>Plant protein	1.self-administered questionnaires<br>2. fasting blood glucose measurements<br>3. 2-h oral glucose tolerance tests<br>4. national registers	Age, examination year, energy intake, marital status, income, use of hypertension medication, family history of diabetes, pack-years of smoking, education, leisure-time physical activity, serum ferritin, alcohol intake, glycemic index, dietary intakes of fiber, Mg, coffee, cholesterol, SFA, MUFA, PUFA and trans-fatty acids, BMI, fasting plasma glucose and fasting serum insulin
Malik	2016	NHS	US	20.1	30–55;<br>Women;<br>7214 cases;<br>76874 subjects	61-item FFQ,<br>131-item FFQ;<br>Total protein,<br>Animal protein,<br>Plant protein	Biennialquestionnaire were mailed a supplementary questionnaire about symptoms andtreatment	Family history of diabetes, smoking, alcohol intake, physical activity, race/ ethnicity, total energyintake, postmenopausal hormone use, percentages of energy from trans- fat, saturated fat, monounsaturated fat, polyunsaturated fat, dietary cholesterol, dietary fiber, glycemic index, percentage of energy derived from animal protein and vegetable protein, BMI
		NHS II		20.1	24–42;<br>Women;<br>5032 cases;<br>88262 subjects	131-item FFQ;<br>Total protein,<br>Animal protein,<br>Plant protein		
		HPFS		20	40–75<br>Men;<br>3334 cases<br>41349 subjects	131-item FFQ;<br>Total protein,<br>Animal protein,<br>Plant protein		

Shang	2016	MCCS	Australia	11.7	53.5±8.4; 38.3%men; 929 cases; 21523 subjects	121-item FFQ; Total protein, Animal protein, Plant protein	Self-reported	Age, sex, ethnicity, socioeconomic status, physical activity, smoking, alcohol intake, glycemic index, consumption of energy, fiber, SFA, MUFA, PUFA , trans fat, animal , plant protein intakes, plasma glucose, blood pressure, BMI
Ericson	2013	MDC	Sweden	12	45–74; 38.2%men; 1709 cases; 26725 subjects	(1) a 7-d menu book (2) a168-item questionnaire (3) a 45-min Interview; Total protein	(1) the Regional Diabetes2000 Register of Scania (2) the MalmoHbA1c Register (3)the Swedish National Diabetes Register	age, method version, season, total energy, education, smoking, alcohol intake, leisure-time physical activity and BMI.
Sluijs	2010	EPIC-NL	Europe	10.1	21-64; 25.6%men; 918 cases; 38094 subjects	A self-administered FFQ containing 79 main food items, validated against 12 24-h dietary recalls; Total protein, Animal protein, Plant protein	Self-report hospital diagnoses	Sex, age atrecruitment, energy-adjusted intake of saturated fat,monounsaturated fat, polyunsaturated fat, cholesterol, vitamin E, magnesium, fiber, glycemic load , energy-adjusted alcohol consumption, physical activity , mean systolic, diastolic blood pressure , education level, parental history of diabetes, BMI, waist circumference
Song	2004	WHS	USA	10	≥ 45; 0%men; 1558 cases; 32688 subjects	A validated SFFQ that inquired about the average use of 131 foods and beverages; Animal protein, Plant protein	By asking women to report these items on annual follow-up questionnaires	Age, BMI, total energy intake, smoking, exercise,alcohol use, family history of diabetes,dietary intakes of fiber intake, glycemic load,magnesium, total fat.

Supplementary table3 Characteristics of included studies of meat consumption and type 2 diabetes

First Author	Publication year	Study name	Country	Follow-up time (year)	Population	Method of dietary exposure assessment and type of meat	Method of diabetes assessment	Adjustment variables
Chen	2019	RS	Netherlands	7.2	≥ 45; 41.4%men; 643 cases, 6813 subjects	170-item FFQ, 389-item FFQ; Fish	1.Fastingblood glucose concentration of7.0mmol/orhigher 2.Non-fastinblood glucose concentration of11.1mmol/L or	Total fat intake, total energy intake, alcohol intake, age, sex,smoking status, education level, diet quality score,physical activity and family history of diabetes,time of WCmeasurements, longitudinal WC, the

							higher<br>3. The use ofhypo-glycemic drugs	interaction between protein intake and longitudinal WC
Jeon	2018	the community-based Ansung-Ansan cohort	Korea	7.3	40-69;<br>47.6%men;<br>1171 cases;<br>8565 subjects	SQFFQ;<br>Fish	Biennial questionnaires, Health examination andclinical tests	Age, sex, body mass index, residential area, education level, household income, physical activity, alcohol consumption, smokingstatus,history of hypertension, family history of type 2 diabetes, use of antihypertensive medication, use of dietary supplements, intakes of vegetables, fruits, red meat, processed meat, soft drinks, coffee, and tea
Jakyung	2018	KoGES	Korea	10	40–69;<br>47.4%men;<br>668 cases;<br>8618 subjects	103-item semi-quantitative food frequency questionnaire;<br>Processed meat	biennial questionnaire-based interview	Age, sex, educational level, monthly household income, residential area, smoking, physical activity, BMI, alcohol intake, energy intake, consumption levels of dietary fat, crude fiber, sodium, fruit and vegetable, current use of antihypertensive and antihyperlipidemic medication
Talaei	2017	SCHS	China	10.9	45–74;<br>42.7 %men;<br>5207 cases,<br>45426 subjects	165-item semi-quantitativefood frequency questionnaire;<br>red meat, poultry;<br>fish	self-reported	Age, sex, dialect, year of interview, educational level, body mass index, physical activity level, smoking status, alcohol use, baseline history of self-reported hypertension, adherence to the vegetable-, fruit-, and soy-rich dietary pattern, total energy intake, heme iron intake
Isanejad	2017	WHI	USA	15	50 – 79;<br>0%men;<br>11242cases;<br>74155 subjects	122-items FFQ;<br>Red meat, Processed meat,<br>Poultry;<br>Fish	Self-report at each semiannual contact when participants were asked by self-administered medical history update questionnaire	Age, ethnicity, education, income, history of CHD, current smoking, current alcohol use, physical activity, hypertension, family history of diabetes, hormone use, glycaemic load, glycaemic index, total energy intake, BMI
Virtanen	2017	KIHD	Finland	19.3	53.1±5.2;<br>100%men;<br>432 cases;<br>2332 subjects	4-d dietary records;<br>Red meat, Processed meat;<br>Fish	Self-administered questionnaires, fasting blood glucose measurements, 2-h oral glucose tolerance tests, national registers	Age, examination year, energy intake, marital status, income, use of hypertension medication, family history of diabetes, pack-years of smoking, education, leisure-time physical activity, serum ferritin, alcohol intake, glycaemic index, and dietary intakes of fibre, Mg, coffee, cholesterol, and SFA, MUFA, PUFA and trans-fatty acids, BMI, fasting plasma glucose and fasting serum insulin
Wallin	2017	COSM	UK	15	45–79;<br>100%men;<br>3624 cases;<br>35583 subjects	96-itemFFQ;<br>fish	Linkage of the study cohort with the Swedish National Diabetes Register (NDR) and the Swedish National Patient	Age, body mass index, physical activity, education, cigarette smoking, total energy intake, intake of alcohol and DASH diet component score ,dietary exposure to polychlorinated

							Register (NPR)	biphenyls and methyl mercury
MariSanchis	2016	SUN Project	Spain	8.84	20-90; 39%men; 146 cases; 18527 subjects	136-items FFQ; Processed meat	Participants reported any medical diagnosis of diabetes at baseline and in each of the follow-up questionnaires.	Age, sex, physical activity, total energy intake, baseline body mass index , family history of diabetes, prevalent hyper-cholesterolemia, prevalent hypertension, dietary fiber intake , sugar-sweetened beverages consumption , smoking status, caffeine intake , glycemic index, adherence to Mediterranean dietary pattern, prevalent cardiovascular disease, prevalent cancer
Ericson	2013	MDC	Sweden	12	45–74; 38.8%men; 1709 cases; 26725 subjects	A 7-d menu book; a 168-item questionnaire a 45-min interview; Red meat, Processed meat, Fish, Poultry	Used information on the date of diagnosis from the registers prioritised in the following order: (1) the Regional Diabetes2000 Register of Scania (2) the Malmo HbA1c Register (3)the Swedish National Diabetes Register	Age, method version, season, total energy,education, smoking, alcohol intake, leisure-time physical activity and BMI.
Kurotani	2013	JPHC	Japan	5	45-75; 42.9%men; 1178cases; 63849 subjects	147- item FFQ; Red meat, Processed meat, Poultry	Self-administered questionnaire	Age, public health centre area, BMI, smoking status, alcohol consumption, total physical activity, the history of hypertension, coffeeconsumption, the family history of diabetes, Mg intake, Ca intake, rice intake, fish intake, vegetable intake, soft drink consumption, energy intake.
Ruesten	2013	Germany	The EPIC-Potsdam study	8	35–65; \ 837 cases; 23531 subjects	Semi-quantitative 148-itemFFQ ; Red meat, Processed meat, Fish, Poultry	Self-reports of the respectivecondition , disease-relevant medication or reasons for a reported change in diet; record linkages	Age, sex, smoking status, pack-years of smoking, alcohol consumption, leisure-time physical activity, BMI, waist-to-hip ratio, prevalent hypertension at baseline, history of high blood lipid levels at baseline, education, vitamin supplementation, total energy intake
Lajous	2012	E3N study	France	13.8	\ 0%men; 1369 cases; 66118 subjects	A validated self-administered dietary questionnaire ; Processed meat	Self-reports, supplementary questionnaires, and drug reimbursement information	Education, residence in the Mediterranean, BMI, smoking, parental history of diabetes, physical activity, hormone replacement therapy, hypertension, hyper-cholesterolemia, n-3 polyunsaturated fatty acid , carbohydrates , fiber, coffee, fruits, vegetables
Amanda	2012	SHFS	USA	8	18.0–74.9; 39%men; 243 cases;	An interviewer-administered	Use of insulin or oral antidiabetic medication or by a	Age, sex, site, total calories/d, education, smoking, alcohol, family

					2001 subjects	Block119-item FFQ; Processed meat	fastingplasma glucoseconcentration ≥126 mg/dL at the follow-up exam in 2007–2009	history of diabetes, pedometer-determined physical activity, fiber from grains and glycemic load, BMI
Geertruida	2012	RS	Nether-lands	12.4	≥55; 42.9%men; 456 cases; 4366 subjects	170 food items FFQ; Red meat, Processed meat, Poultry	Records of general practitioners' (including laboratory glucose measurements), hospital discharge letters, and serum glucose measurements	Age, sex, smoking, diet prescription, family history of diabetes, intake of energy, energy-adjustedcarbohydrates ,energy-Adjustedpolyunsaturated fatty acids, energy-adjusted fiber, energy-adjusted milk, energy-adjusted cheese, soya, fish, alcohol, tea, intake of processed meat and poultry
Pan	2011	NHS	USA	20.1	30–55; 0%men; 8253 cases; 100208 subjects	61\131-item FFQ; Red meat, Processed meat	Biennial questionnaire weremailed a supplementary questionnaire aboutsymptoms and treatment	Age, alcohol consumption , physical activity level, smoking status , race , menopausal status and hormone use in women, family history of diabetes, history of hypertension and hypercholesterolemia, quintiles of total calories, dietary score, a BMI category
		NHS II		20.1	24–42; 0%men; 3068 cases; 67969 subjects	131-item FFQ; Red meat, Processed meat		
		HPFS		20	40–75; 100%men; 2438 cases; 32649 subjects	131-item FFQ; Red meat, Processed meat		
Steinbrecher	2011	MEC	USA	13.5	45-75; 48.0%men; 8587 cases; 75512 subjects	A validated quantitative food frequency questionnaire ; Red meat, Processed meat, Poultry	Self-report in a follow-up questionnaire; a medication questionnaire; a linkagein 2007 with the two major health plans	Ethnicity, education, BMI, physical activity and total calorie intake, stratified by age at cohort entry
Nanri	2011	JPHC	Japan	5	45-75; 42.9%men; 971 cases; 52680 subjects	147- item FFQ; Fish	Self-administered questionnaire	Age, study area, BMI, smoking status , alcohol consumption , family history of diabetes mellitus , total physical activity , history of hypertension , total energy intake , coffee consumption, intakes of calcium, magnesium, dietary fiber, vegetables, fruit, meat, rice
Villegas	2011	SWHS	China	10	40-70; 0%men; 3034 cases; 64193 subjects	A validated FFQ questionnaire ; Fish	Self-reported	Age, energy intake, waist-to-hip ratio, BMI, smoking, alcohol consumption, physical activity,income level, educational level, occupation, family history of diabetes, hypertension, dietary pattern
		SMHS		6	40-74; 100%men; 900 cases; 51963 subjects			
Djousse	2011	WHS	USA	12.4	≥45; 0% men; 2370 cases; 36328	128-food-frequency questionnaire ;	By annualfollowup questionnaires, validated byusing the ADA criteria,	age, BMI, parental history of diabetes, smoking, exercise, alcoholintake, menopausal status, red-meat intake,

					subjects	Fish	obtainingadditional information with a telephone interview andsupplemental questionnaire	quintiles of energyintake, linoleic acid, a-linolenic acid, dietary magnesium, trans fat, saturated fat, cereal fiber, and glycemic index
Satu	2010	ATBC	Finland	12	50-69; 100%men; 1098 cases; 25943 subjects	276 food items and mixed dishes, a validated self-administ ered FFQ; Red meat, Processed meat, Poultry	A medical certificate from the attendingphysician, the certificate of every case is verified to fulfill the diagnostic criteria (blood glucose permanently 7.0 mmol/l or higher after dietary treatment) for diabetes at the Social Insurance Institution	Age, intervention group, body mass index, number of cigarettes smoked daily, smoking years, systolic blood pressure, diastolic blood pressure, serum total cholesterol, serum high-density lipoprotein cholesterol, leisure-time physical activity, intakes of alcohol and energy, consumption of fruit, vegetables, rye, milk and coffee
Patel	2009	EPIC-Norfolk Study	United Kingdom	10.2	40–79; 44.7%men; 725 cases; 21984 subjects	130-item semi-quantita tive FFQ; Fish	Self-report confirmed by record linkage with several databases	Age, sex, family history of diabetes,smoking, education level, physical activity, total energy intake, alcohol intake, plasma vitamin C, BMI, waist circumference
Geertruida	2009	RS	Nether-lands	12.4	≥55; 42.9%men; 463 cases; 4472 subjects	170 food items FFQ; Fish	Records of general practitioners' (laboratory glucose measurements), hospital discharge letters, and serum glucosemeasurements	Age, sex, smoking, education level, intake of energy, alcohol, trans fatty acids, fiber
Kaushik	2009	NHS	USA	15.1	30–55; 0%men; 4159 cases; 94159 subjects	61-item FFQ; 131-item FFQ; Fish	Biennial questionnaire weremailed a supplementary questionnaire aboutsymptoms and treatment	Smoking, alcohol consumption,physical activity, family history of diabetes mellitus, BMI, intakes of saturated fat, trans fats, linolenic acid, linoleic acid, caffeine, cereal fiber; glycemic index, calories,menopausal status, postmenopausal hormone use
		NHS II		15.1	26–46; 0%men; 2728 cases; 96682 subjects	131-item FFQ; Fish	Biennial questionnaire weremailed a supplementary questionnaire aboutsymptoms and treatment	smoking, alcohol consumption,physical activity, family history of diabetes mellitus, BMI, intakes of saturated fat, trans fats, linolenic acid, linoleic acid, caffeine, cereal fiber; glycemic index, calories,use of hormone replacement therapy and oral contraceptive use
		HPFS		15.1	39–78; 100%men; 2493 cases; 44902 subjects	131-item FFQ; Fish	Biennial questionnaire weremailed a supplementary questionnaire aboutsymptoms and treatment	smoking, alcohol consumption,physical activity, family history of diabetes mellitus, BMI, intakes of saturated fat, trans fats, linolenic acid, linoleic acid, caffeine, cereal fiber, glycemic index, calories

Vang	2008	AMS and AHS	USA	17	45–88; 58.3%men; 531 cases; 8401 subjects	Semi-quantitative questionnaire; Fish	Self-report on annual follow-up questionnaires	Age, sex
Villegas	2006	SWHS	China	4.6	40-70; 0%men; 1969 cases; 70609 subjects	A validated FFQ; Red meat, Processed meat, Poultry	Self-reported	Age, kcals/day, BMI, WHR, smoking, alcohol consumption, physical activity, vegetable intake, income level, education level, occupationstatus, hypertension, chronic disease
Montonen	2005	Finnish Mobile Clinic Health Examination Survey	Finland	23	40–69; 53.1%men; 383 cases; 4304 subjects	Dietary history interviewa questionnaire form listing over 100fooditems and mixed dishes ; Red meat, Processed meat, Poultry	Identified from the SocialInsurance Institution's nationwide register of persons receiving drug reimbursement	Age, sex, geographicarea, BMI, smoking, familyhistory of diabetes, total energyintake
Song	2004	WHS	USA	10	≥45; 0%men; 1543 cases; 39876 subjects	a validated SFFQ that inquired about the average use of 131 foods and beverages; Red meat, Processed meat	By asking women to report these items on annual follow-up questionnaires; validated byusing the ADA criteria; obtaining additional information with a telephone interview andsupplemental questionnaire	Age, BMI, total energy intake, smoking, exercise, alcohol use, family history of diabetes,dietary intakes of fiber intake, glycemic load, magnesium, total fat
Dam	2002	The Health Professionals Follow-up Study	USA	12	40–75; 100%men; 1320 cases; 42504subjects	Asemi-quantitative FFQ that inquired about the average use of 130 foods and beverages; Processed meat	Self-reported diabetes was confirmed by a supplementary questionnaire, and validation with medical records	Age, total energy intake, time period, physical activity, cigarette smoking, alcohol consumption,hyper-cholesterolemia, hypertension,family history of type 2 diabetes, intake of cereal fiber and magnesium, BMI

Supplementary table4 Characteristics of included studies of total dairy consumption and type 2 diabetes

First Author	Publication year	Study name	Country	Follow-up time (year)	Population	Method of dietary exposure assessment and type of dairy	Method of diabetes assessment	Adjustment variables
Talaei	2018	SCHS	China	12	45–74; 42.7 %men; 5207 cases,	165-item semi-quantitative FFQ;	Self-reported	Age, sex, dialect, year of interview, educational level, body mass index, physical

					45426 subjects	Milk		activity, smoking status, alcohol use, baseline history of self-reported hypertension, total energy intake, vegetable, fruit, soy-rich pattern, dim sum, meat-rich pattern, coffee, soda
Jeon	2018	The community-based Ansung-Ansan cohort	Korea	7.3	51.7 ± 0.1; 47.6%men; 1171 cases; 8558 subjects	SQFFQ; Milk, Yogurt	Biennial questionnaires, health examinations, clinical tests	Age, sex, body mass index, residential area, education level, household income, physical activity, alcohol consumption, smoking status,history of hypertension, family history of type 2 diabetes, use ofantihypertensive medication, use of dietary supplements, intakes of vegetables, fruits, red meat, processed meat, soft drinks, coffee, tea
Virtanen	2017	KIHD	Finland	19.3	53.1±5.2; 100%men; 432 cases; 2332 subjects	4-d dietary records; Milk	Self-administered questionnaires, fasting blood glucose measurements, 2-h oral glucose tolerance tests, national registers	Age, examination year, energy intake, marital status, income, use of hypertension medication, family history of diabetes, pack-years of smoking, education, leisure-time physical activity, serum ferritin, alcohol intake, glycaemic index, and dietary intakes of fibre, Mg, coffee, cholesterol, and SFA, MUFA, PUFA and trans-fatty acids, BMI, fasting plasma glucose and fasting serum insulin
Brouwer-Brolsma	2016	RS	Netherlands	9.5	65.1±6.7; 40%men; 393 cases; 2974 subjects	170 food items FFQ; Milk, Yogurt	Records of general practitioners' (including laboratory glucose measurements), hospital discharge letters, and serum glucose measurements	Age, sex, alcohol , smoking, education, physical activity , BMI, total energy intake, energy adjusted meat intake, energy-adjusted fish intake, potential intermediates
Andrés	2016	PREDIMED	Spain	4.1	66.6±6.6; 38.4% men; 270 cases; 3454 subjects	137-item semi-quantitative FFQ; Milk, Yogurt	Clinical diagnosis or use of antidiabetic medication	Age, sex, BMI, dietary intervention group, leisure time physical activity, educational level , smoking, hypertension or antihypertensive use ,fasting glucose, HDL-cholesterol, triglyceride concentrations, cumulative average consumption of dietary variables in energy-adjusted quintiles, alcohol
Ericson	2015	MDC	Sweden	14	45–74; 38.8%men; 2860 cases; 24070 subjects	A 7-d menu book, a 168-item questionnaire, a 45-min interview; Milk	Used information on the date of diagnosis from the registers prioritised in the following order: (1) the Regional Diabetes2000 Register of Scania (2) the Malmo HbA1c Register	Age, sex, method version,season, education, BMI, leisure-time physical activity,smoking, intakes of total energy and alcohol

							(3)the Swedish National Diabetes Register	
Connor	2014	EPIC-Norfolk Study	UK	11	40–79; 44%men; 752 cases; 4126 subjects	130-item semi-quantitative FFQ, 7-d food diary; Milk, Yogurt	Self-report confirmed by record linkage with several databases	Age, sex, BMI, family history of diabetes, smoking, alcohol, physical activity, social class, education level, energy, fiber, fruit,vegetables, red meat, processed meat, coffee intake
Geng	2014	NHAP CS	China	6	50–70; 41%men; 507 cases; 2091 subjects	74-item FFQ; Milk	Self-report, Useof any oral hypoglycemic medication or insulin, or fasting glucose ≥7.0mmol/L	Age, sex, region, residence, smoking, family history of diabetes, BMI, dietary fiber intake, changes in BMI and waistline
Sabita	2013	Whitehall IIstudy	UK	10	56±6.1; 72%men; 273 cases; 4186 subjects	Validated 114 items FFQ; Milk, Yogurt	Self-report of doctor's diagnosis, initiation of antidiabeticmedication, a 2-h 75-g oral-glucose tolerancetest	Age, sex, ethnicity,employment grade, smoking, alcohol intake, BMI, physical activity,family history of coronary heartdisease/ hypertension, fruit,vegetables, bread, meat, fish, coffee, tea, total energy intake
Struijk	2013	Inter99 Study	Denmark	5	30-60; 48%men; 214 cases; 5232 subjects	Validated 198-item FFQ; Milk	Fasting plasma glucose ≥7.0 mmol/L and/or 2-h plasma glucose ≥11.1 mmol/L based on 1oral-glucosetolerance test	Age, sex, intervention group, diabetes family history, education level, physical activity, smoking status, intake of alcohol, wholegrain cereal, meat, fish, coffee, tea, fruit, vegetables, energy, change in diet form baseline to 5-y follow-up, waist circumference
Grantham	2013	AusDiab	Australia	5	25-88; 45%men; 209 cases; 5582 subjects	121-itemFFQ; yogurt	Fasting plasma glucose ≥7.0 mmol/L or 2-h postload plasma glucose ≥11.1 mmol/L or treatment with insulin or oral hypoglycemic agents	Age, sex, energy intake, family history of diabetes,education level, level of physical activity, smoking status, triglycerides, HDLcholesterol, systolic blood pressure, waist circumference, hip circumference
Kirii	2009	JPHC	Japan	5	45-75; 42.9% men; 1114 cases; 59796 subjects	147-item FFQ; Milk, Yogurt	Self-administered questionnaire	Age , area , BMI, family history of diabetes mellitus, smoking status , alcohol intake, history of hypertension, exercise frequency, consumption of coffee , energy-adjusted magnesium, total energy
Vang	2008	AMS and AHS	USA	17	45–88; 61.1% men; 539 cases; 8401 subjects	Semi-quantitative questionnaire; Milk	Self-report on annual follow-up questionnaires	Age, sex
Liu	2006	WHS	USA	10	≥ 45; 0% men; 1603 cases; 39876 subjects	A validated SFFQ that inquired about the average use of 131	By asking women to report these items on annual follow-up Questionnaires; validated by	Total energy intake,randomized-treatment assignment, age, family history of diabetes, smoking status, BMI, hypercholesterolemia,

						foods and beverages; Yogurt	using the ADAcriteria; primarily obtainadditional information with a telephone interview andsupplemental questionnaire	hypertension, physicalactivity,hormones, alcohol consumption, dietary intakes,fibers, total fat, dietary glycemic load,quintiles of dietary, calcium, vitamin D, magnesium.
Choi	2005	The Health Professionals Follow-up Study	USA	12	40-75; 100% men; 1243 cases; 37726 subjects	A semi-quantitative FFQ that inquired about the average use of about 130 foods and beverages; Milk, Yogurt	Self-reported diabetes was confirmed by a supplementary questionnaire, and validation with medical records	Age , total energy intake, biennial follow-up time, family history of diabetes, smoking status , BMI, hypercholesterolemia , hypertension, physical activity , alcohol intake,cereal fiber intake, trans-fat intake, ratio of polyunsaturated to saturated fat, glycemic load
Montonen	2005	Finnish Mobile Clinic Health Examination Survey	Finland	23	40–69; 53.1%men; 383 cases; 4304 subjects	Dietary history interviewa questionnaire form listing over 100 food items and mixed dishes; Milk	Identified from the SocialInsurance Institution's nationwide register of persons receivingdrug reimbursement	Age, sex, geographic area, BMI, smoking, family history of diabetes, total energyintake

Supplementary table5 Characteristics of included studies of soy consumption and type 2 diabetes

First Author	Publication year	Study name	Country	Follow-up time (year)	Population	Method of dietary exposure assessment	Method of diabetes assessment	Adjustment variables
Chen	2019	RS	Netherlands	7.2	65.4± 11.3; 41.4%men; 643 cases; 6813 subjects	Semi-quantitative 170-item FFQ, semi-quantitative 389-item FFQ	1. Fasting blood glucose concentration of 7.0 mmol/L or higher; 2. Non-fasting blood glucose concentration of 11.1mmol/L or higher; 3. The use of blood glucose-lowering medications.	Total fat intake, total energy intake , alcohol intake , age, sex,smoking status, education level, diet quality score, physical activity family history of diabetes,time point of WC measurements, longitudinal WC, the interaction between protein intake and longitudinal WC
Nerea	2018	PREDIMED	Spain	4.3	67 ±6; 38.4% men; 266 cases; 13797 subjects	Semi-quantitative FFQ	A diagnosis reported in the medical charts or on a fasting blood glucose values during routine biochemical analyses	Age, sex, intervention group, cumulative average consumption of alcohol , smoking status, educational level, leisure-time physical activity ,baselinehypertension , hypercholesterolemia, use of antihypertensive medication , use of lipid-lowering drugs and fasting plasma glucose at baseline ,cumulative average of the 13-point screener (excluding legumes) of MedDiet adherence, BMI
Ding	2016	NHS	USA	20.1	30–55; 0%men;	61\131-item FFQ	Biennial questionnaire	Age, alcohol consumption , physical activity level,

					4519 cases; 38885 subjects		weremailed a supplementary questionnaire aboutsymptoms and treatment	smoking status , race , menopausal status, hormone use in women, family history of diabetes, history of hypertension and hyper-cholesterolemia, quintiles of total calories, dietary score, a BMI category
		NHS II		20.1	24–42; 0%men; 3920 cases; 51589 subjects	131-item FFQ		
		HPFS		20	40–75; 100%men; 742cases; 7364 subjects	131-item FFQ		
Ericson	2013	MDC	Sweden	12	45–74; 38.8%men; 1571 cases; 26725 subjects	A 7-d menu book; a 168-item questionnaire a 45-min interview	Used information on the date of diagnosis from the registersprioritised in the following order:(1) the Regional Diabetes 2000 Register of Scania(2) the Malmo HbA1c Register(3)the Swedish National Diabetes Register	Age, method version, season, total energy, education, smoking, alcohol intake, leisure-time physical activity, BMI.
Tatsumi	2013	The Saku Study	Japan	2.4	30-70; 100% men; 146 cases; 1738 subjects	Aself-administered questionnaire	Fasting hyperglycemia (FPG levels≥7.0 mmol/L), and/or postload hyperglycemia (2-h PGlevels ≥11.1 mmol/L), receiving medical treatment for type 2 diabetes mellitus	Age, body mass index, alcohol consumption, smoking status,physical activity, family history of diabetes, green vegetable intake and fruit intake
Ruesten	2013	German y	The EPIC-Po tsdam study	8	35–65; \ 837 cases; 23531 subjects	Semi -quantitative 148-itemFFQ	Self-reports of the respectivecondition , disease-relevant medication or reasons for a reported change in diet;record linkages with the Common Cancer registry and the database of the clinical center of Potsdam	Age, sex, smoking status, pack-years of smoking, alcohol consumption, leisure-time physical activity, BMI, waist-to-hip ratio, prevalent hypertension at baseline, history of high blood lipid levels at baseline, education, vitamin supplementation, total energy intake
Mueller	2012	SCHS	China	5.7	45–74; 42.7%men; 2252 cases; 246898 subjects	165-item semi-quantita tive food frequency questionnaire	Self-reported	Age, sex, dialect, year of interview, soybean drink , educational level , smoking status , alcohol use , any physical activity , baseline hypertensive , calcium , carbohydrate , polyunsaturated fatty acid, non-soy protein, total energy, sweetened soybean drink and tofu, body mass index
Morimot o	2011	MEC	USA	14	45-75; 53.1%men; 8564 cases; 26068 subjects	A validated quantitative food frequency questionnaire (FFQ)	Self-report in a follow-up questionnaire; a medication questionnaire; by a linkage in 2007 with the	Ethnicity, BMI, physical activity, education, total energy, smoking status, alcohol, dietary fiber, processed red meat intake

							two major health plans	
Nanri	2010	JPHC	Japan	5	45-75; 42.9%men; 1114 cases; 59791 subjects	147- item FFQ	Self-administered questionnaire	Age, study area, BMI , smoking habit, alcohol consumption , family history of diabetes mellitus, leisure time physical activity, history of hypertension , coffee consumption , green tea consumption, , vegetable intake, fiber intake, fish intake , and total energy intake
Villegas	2008	SWHS	China	4.6	40-70; 0%men; 1605 cases; 70609 subjects	A validated food-frequency questionnaire (FFQ)	Self-reported	Age, energy intake, BMI, waist-to-hip ratio, smoking, alcohol consumption, vegetable intake, fiber, physical activity, income level, education level, occupation, and hypertension.

Supplementary table6Characteristics of included studies of egg consumption and type 2 diabetes

First Author	Publication year	Study name	Country	Follow-up time (year)	Population	Method of dietary exposure assessment	Method of diabetes assessment	Adjustment variables
Jing Guo	2018	CAPS	England	22.8	45–59; 100%men; 120 cases; 1687 subjects	FFQ	Self-reported from questionnaires	Age,BMI,total energy intake, alcohol consumption, smoking, energy expenditure, social class,family history of myocardial infarction,sugar intake, fruit consumption , red meat consumption and fibre (cereal and vegetable sources)
Sabaté	2018	AHS-2	United States and Canada	5.3	44.3-71.3; 35.2%men; 2594cases; 52718 subjects	FFQ	HHQ3, HHQ5	Age, race, gender, energy intake, television hours,sleep hours,smoking, exercise, refined grains,vegetables, coffee, dairy, soy, nuts/seeds, fruits, fish, egg intake, meat intake and BMI
Jieul Lee	2018	KoGES	Korea	9.2	40–69; 47.4%men; 857 cases; 7002 subjects	103-item semi-quantitative food frequency questionnaire	Fasting glucose concentration ≥126 mg/dL; the current use of glucose-lowering medications, or the use of insulin injection based on themodified WHO criteria;Aself-reported physician’sdiagnosis	Age, BMI, residential location, education level, household income, smoking status, alcohol intake, physical activity, intake of total energy, cholesterol, fiber, meat, fish, vegetables, fruit, and dairy
Virtanen	2017	KIHD	Finland	19.3	53.1±5.2; 100%men; 432 cases; 2332	4-d dietary records	Self-administered questionnaires, fasting blood glucose	Age, examination year, energy intake, marital status, income, use of hypertension medication, family history of

					subjects		measurements,<br>2-h oral glucose tolerance tests,<br>national registers	diabetes, pack-years of smoking, education, leisure-time physical activity, serum ferritin, alcohol intake, glycaemic index, and dietary intakes of fibre, Mg, coffee, cholesterol, and SFA, MUFA, PUFA and trans-fatty acids, BMI, fasting plasma glucose and fasting serum insulin
Wallin	2016	COSM	UK	15	45–79;<br>100%men;<br>4173 cases;<br>39610 subjects	96-itemFFQ	Linkage of the study cohort with the Swedish National Diabetes Register (NDR) and the Swedish National Patient Register (NPR)	Age, BMI, physical activity; education, cigarette smoking total energy intake , intake of alcohol, history of cardiovascular disease at baseline,coffee consumption, and intakes of red meat, processed meat, fish,fruit, vegetables, white bread, caviar,sweet buns/biscuits, fibre
Djoussé	2016	JHC	USA	7.3	21-95;<br>36% men;<br>531 cases;<br>3564 subjects	158-item FFQ	Fasting glucose ≥126 mg/dL, hemoglobin A1C ≥ 6.5%,<br>or current use of insulin or oral hypoglycemic	Age, sex, smoking, alcohol, BMI, physical activity score, education,energy intake, red meat (including bacon), fiber, dietary magnesium, fruit/vegetables, trans fat, waist circumference, history of hypertension, history of CVD
Lajous	2015	E3N	France	14	43-70;<br>0% men;<br>1803 cases;<br>65364 subjects	208-item self-administ ered diet history questionnaire	Self-reports, supplementary questionnaires and drug reimbursement information	Age, education, BMI, smoking, physical activity, menopause,<br>hormone replacement therapy,hypertension, hyper-cholesterolaemia, energy,alcohol, processed red meat,coffee, fruits, vegetables,sugar-sweetened artificially sweetened drinks
Ericson	2015	MDC	Sweden	14	45–74;<br>38.8%men;<br>2860 cases;<br>24070 Subjects (2860)	A 7-d menu book;<br>a 168-item questionnaire<br>a 45-min interview	Used information on the date of diagnosis from the registersprioritised in the following order:(1) the Regional Diabetes 2000 Register of Scania(2) the Malmo HbA1c Register(3)the Swedish National Diabetes Register	Age, sex, method version, season, education, BMI, leisure-time physical activity, smoking, intakes of totalenergy and alcohol
Kurotani	2014	JPHC	Japan	5	45-75;<br>42.9%men;<br>1165cases;<br>63466 subjects	147- item FFQ	Self-administered questionnaire	Age, public health centre, area,BMI, smoking status, alcohol consumption, total physical activity levels, history of hypertension and family history of diabetes, Mg intake, Ca intake, coffee consumption, rice intake,fish and shellfish intake, meat intake, vegetable intake,<br>soft drink intake and energy intake.
Zazpe	2013	SUN Project	Spain	6.6	20–90;<br>40.4% men;	Semi -quantitative	Whether received a medical diagnosis	Age, sex, total energy intake, adherence to the

					91 cases; 15956 subjects	FFQwith 136 items	of diabetes	Mediterranean food pattern, alcohol intake, baseline BMI, smoking status, physical activity during leisure time,family history of diabetes, self-reported ECV, self-reported hypertension, self-reported hypercholesterolemia
Ruesten	2013	German y	The EPIC-Po tsdam study	8	35–65; \ 837 cases; 23531 subjects	Semi -quantitative 148-itemFFQ	Self-reports of the respectivecondition , disease-relevant medication or reasons for a reported change in diet;record linkages with the Common Cancer registry and the database of the clinical center of Potsdam	Age, sex, smoking status, pack-years of smoking, alcohol consumption, leisure-time physical activity, BMI, waist-to-hip ratio, prevalent hypertension at baseline, history of high blood lipid levels at baseline, education, vitamin supplementation, total energy intake
Djoussé	2010	CHS	USA	11.3	65–98; 42.8% men; 313 cases; 5888 subjects	99-item picture-sort version of the National Cancer Institute FFQ	Newuse of insulin or oral hypoglycemic agents or a fasting glucose concentration≥7 mmol/L or a nonfasting glucose concentration of ≥11.1 mmol/L	Age, race, field centre, BMI, physical activity, smoking, intakes of alcohol and cereal fibre
Djoussé	2009	PHS 1	USA	20	39.7–85.9; 100%men; 1921 cases; 20703 subjects	Self-reported using asimple abbreviated semi-quantitative FFQ	Self-report on annual follow-up questionnaires	Age, BMI, vigorous exercise, smoking, history of hyper-cholesterolaemia and hypertension, alcohol intake
		WHS		11.7	38.7–89.9; 0%men; 2076 cases; 36295 subjects			Age, BMI, exercise, smoking,family history of diabetes, history of hypertension,hyper-cholester olaemia, intakes of energy, alcohol, red meat, fruits and vegetables, saturatedfatty acids, trans-fatty acids,PUFAs
Vang	2008	AMS and AHS	USA	17	45–88; 58.3%men; 535cases; 8401 subjects	Semi -quantitative questionnaire	Self-report on annual follow-up questionnaires	Age, sex
Monto-n en	2005	Finnish Mobile Clinic Health Examin ation Survey	Finland	23	40–69; 53.1%men; 383 cases; 4304 subjects	Dietaryhistor y interviewa questionnaire form listing over 100 food items and mixed dishes common in the Finnish diet	identified from the SocialInsurance Institution’s nationwide register of persons receivingdrug reimbursement	age, sex, geographicarea, BMI, smoking, familyhistory of diabetes, total energyintake

Supplementary table7: Quality assessment of cohort studies included in meta-analysis (Newcastle-Ottawa Quality Assessment Scale)

First author, year	Cohort	Selection					Comparability			Outcome				Total (9Max)
		Q 1	Q 2	Q 3	Q 4	Sub-Total	Q1 A*	Q1 B**	Sub-Total	Q 1	Q 2	Q 3	Sub-Total	
Chen 2019[1]	RS-I	1	1	1	1	4	1	1	2	1	1	0	2	8
	RS-II	1	1	1	1	4	1	1	2	1	1	0	2	8
	RS-III	1	1	1	1	4	1	0	1	1	1	0	2	7
Talaei2018[2]	SCHS	1	1	1	1	4	1	0	1	0	1	1	2	7
Jeon 2019[3]	CBAAC	1	1	1	1	4	0	1	1	1	1	1	3	8
Talaei 2017[4]	SCHS	1	1	1	0	3	1	0	1	0	1	1	2	6
Nerea 2018[5]	PREDIMED	1	1	1	1	4	0	0	0	1	0	1	2	6
Lee 2018[6]	KoGES	1	1	1	1	4	0	1	1	1	1	0	2	8
Son 2018[7]	KoGES	1	1	1	1	4	0	0	0	1	1	1	3	7
Joan 2018[8]	AHS-2	1	1	1	1	4	1	0	1	1	1	0	2	7
Isanejad 2017[9]	WHI	1	1	1	1	4	1	1	2	0	1	1	2	8
Virtanen 2017[10]	KIHD	1	1	1	1	4	1	1	2	1	1	1	3	9
Wallin2017[11]	COSM	1	1	1	1	4	1	0	1	1	1	1	3	8
Malik 2016[12]	NHS	0	1	1	1	3	0	1	1	0	1	1	2	6
	NHS II	0	1	1	1	3	0	1	1	0	1	1	2	6
	HPFS	0	1	1	1	3	0	1	1	0	1	1	2	6
MariSanchis 2016[13]	SUN Project	0	1	1	1	3	1	1	2	0	1	1	2	7
BrouwerBrolsma 2016[14]	the Rotterdam Study	1	1	1	1	4	1	0	1	1	1	1	3	8
Shang 2016[15]	MCCS	1	1	1	1	4	1	0	1	0	1	1	2	7
Andrés 2016[16]	PREDIMED	0	1	1	1	3	1	0	1	1	0	1	2	6
Ding 2016[17]	NHS	0	1	1	1	3	0	1	1	0	1	1	2	6
	NHS II	0	1	1	1	3	0	1	1	0	1	1	2	6
	HPFS	0	1	1	1	3	0	1	1	0	1	1	2	6
O'Connor 2014[18]	EPIC-Norfolk Study	1	1	1	1	4	1	1	2	0	1	1	2	8
Geng 2014[19]	NHAPC	1	1	1	1	4	0	0	0	0	1	1	2	6
Sabita2013[20]	Whitehall II study	1	1	1	1	4	1	0	1	1	1	1	3	8
Struijk 2013[21]	Inter99 Study	1	1	1	1	4	1	1	2	1	1	0	2	8
Kurotani 2013[22]	JPHC	1	1	1	1	4	1	1	2	0	1	0	1	7
Ericson 2013[23]	MDC	1	1	1	1	4	1	0	1	1	1	0	2	7
Tatsumi 2013[24]	Saku	1	1	1	1	4	0	1	1	1	0	0	1	6
Lajous 2012[25]	E3N	1	1	1	1	4	0	1	1	1	1	1	3	8
Amanda 2012[26]	SHFS	1	1	1	1	4	1	1	2	1	1	0	2	8
Geertruida2012 [27]	the Rotterdam Study	1	1	1	1	4	1	0	1	1	1	1	3	8

Mueller2012[28]	SCHS	1	1	1	0	3	1	0	1	0	1	1	2	6
Grantham 2013[29]	AusDiab	1	1	1	1	4	1	1	2	1	0	0	1	7
Ruesten 2013[30]	EPIC-Potsdam	1	1	1	1	4	1	0	1	1	1	1	3	8
Pan 2011[31]	NHS	0	1	1	1	3	1	1	2	0	1	1	2	7
	NHS II	0	1	1	1	3	1	1	2	0	1	1	2	7
	HPFS	0	1	1	1	3	1	1	2	0	1	1	2	7
Steinbrecher 2011[32]	MEC	1	1	1	1	4	1	0	1	1	1	0	2	7
Nanri 2011[33]	JPHC	1	1	1	1	4	1	1	2	0	1	0	1	7
Villegas 2011[34]	SWHS	1	1	1	1	4	1	1	2	0	1	1	2	8
	SMHS	1	1	1	1	4	1	1	2	0	1	1	2	8
Morimoto 2011[35]	MEC	1	1	1	1	4	0	0	0	1	1	0	2	6
Nanri 2010[36]	JPHC	1	1	1	1	4	1	1	2	0	1	0	1	7
Djousse2011[37]	WHS	1	1	1	1	4	1	1	2	0	1	1	2	8
Satu 2010[38]	ATBC	0	1	1	1	3	1	0	1	0	1	1	2	6
Sluijs 2010[39]	EPIC-NL	1	1	1	1	4	1	1	2	1	1	0	2	8
Kirii 2009[40]	JPHC	1	1	1	1	4	1	1	2	0	1	0	1	7
Pinal 2009[41]	EPIC-NorfolkStudy	1	1	1	1	4	1	1	2	0	1	1	2	8
Geertruida2009 [42]	TheRotterdam Study	1	1	1	1	4	1	0	1	1	1	1	3	8
Kaushik 2009[43]	NHS	0	1	1	1	3	0	1	1	0	1	1	2	6
	NHS II	0	1	1	1	3	0	1	1	0	1	1	2	6
	HPFS	0	1	1	1	3	0	1	1	0	1	1	2	6
Vang 2008[44]	AMS/AHS	1	1	1	1	4	0	0	0	0	1	1	2	6
Villegas 2008[45]	SWHS	1	1	1	1	4	1	0	1	0	1	1	2	7
Liu 2006[46]	WHS	1	1	1	1	4	1	1	2	0	1	1	2	8
Choi 2005[47]	HPFS	0	1	1	1	3	1	1	2	0	1	1	2	7
Zazpe 2013[48]	SUN Project	0	1	1	1	3	1	1	2	0	1	1	2	7
Kurotani 2014[49]	JPHC	1	1	1	1	4	1	1	2	0	1	0	1	7
Djoussé 2016[50]	JHS	1	1	1	1	4	1	0	1	1	1	0	7	7
Guo2018[51]	CAPS	1	1	1	1	4	1	0	1	0	1	0	1	6
Djousse 2010[52]	CHS	1	1	1	1	4	0	0	0	1	1	1	3	7
Djousse 2009[53]	WHS	1	1	1	1	4	1	1	2	0	1	1	2	8
	PHS 1	1	1	1	1	4	0	0	0	0	1	1	2	6
Wallin2016[54]	COSM	1	1	1	1	4	1	0	1	1	1	1	3	8
Ericson 2015[55]	MDC	1	1	1	1	4	1	0	1	1	1	0	2	7
Montonen 2005[56]	FMCHES	1	1	1	1	4	1	0	1	1	1	1	3	8
Villegas 2006[57]	SWHS	1	1	1	1	4	1	0	1	0	1	1	2	7
Song 2004[58]	WHS	1	1	1	1	4	1	1	2	0	1	1	2	8
Dam 2002[59]	HPFS	0	1	1	1	3	1	1	2	0	1	1	2	7
Lajous 2015[60]	E3N	1	1	1	1	4	0	1	1	1	1	1	3	8

Supplementary Table 8 Dose-response meta-analysis for per 5% of energy/day increase in total protein and risk of type 2 diabetes, stratified by high vs. low intake, gender, age, follow-up, geographic location, number of cases

Dietary factor	No of studies	RR	95% CI	I^2 (%)	P
Total protein					
High vs. low	12	1.11	1.05 , 1.16	5.1	0.395
Dose-response	12	1.08	1.05 , 1.11	0.0	0.544
Gender					
Women	3	1.08	1.00 , 1.17	0.0	0.658
Men	3	1.10	1.04 , 1.15	0.0	0.493
Men and women	6	1.14	1.06 , 1.23	0.0	0.587
Age					
<50	2	1.04	0.98 , 1.11	36.8	0.208
≥50	10	1.09	1.06 , 1.13	0.0	0.663
Follow-up					
<10 years	4	1.12	1.00 , 1.24	10.2	0.342
≥10 years	8	1.08	1.05 , 1.11	0.0	0.523
Geographic location					
Europe	8	1.09	1.04 , 1.14	0.0	0.654
America	3	1.07	1.02 , 1.11	30.1	0.239
Australia	1	1.15	1.04 , 1.28	NA	
Number of cases					
<1000	9	1.10	1.05 , 1.14	0.0	0.651
≥1000	3	1.07	1.02 , 1.11	30.1	0.239

Supplementary Table 9 Dose-response meta-analysis for per 5% of energy/day increase in animal protein and risk of type 2 diabetes, stratified by high vs. low intake, gender, age, follow-up, geographic location, number of cases

Dietary factor	No of studies	RR	95% CI	I^2 (%)	P
Animal protein					
High vs. low	11	1.13	1.08 , 1.19	14.3	0.307
Dose-response	11	1.11	1.07 , 1.15	42.7	0.065
Gender					
Women	3	1.08	1.03 , 1.13	80.9	0.005
Men	2	1.18	1.08 , 1.28	0.0	0.749
Men and women	6	1.14	1.05 , 1.24	0.0	0.659
Age					
<50	1	1.06	0.98 , 1.14	NA	
≥50	10	1.13	1.08 , 1.17	42.3	0.076
Follow-up					
<10 years	4	1.10	0.99 , 1.21	0.0	0.814
≥10 years	7	1.11	1.07 , 1.16	63.5	0.012
Geographic location					
Europe	6	1.10	1.01 , 1.21	0.0	0.954
America	4	1.10	1.06 , 1.15	78.1	0.003
Australia	1	1.29	1.07 , 1.55	NA	
Number of cases					
<1000	7	1.14	1.05 , 1.23	0.0	0.773
≥1000	4	1.10	1.06 , 1.15	78.1	0.003

Supplementary Table 10 Dose-response meta-analysis for per 5% of energy/day increase in plant protein and risk of type 2 diabetes, stratified by high vs. low intake, gender, age, follow-up, geographic location, number of cases

Dietary factor	No of studies	RR	95% CI	I² (%)	P
Plant protein					
High vs. low	11	0.93	0.87 , 0.99	0.0	0.479
Dose-response	10	0.85	0.76 , 0.96	41.7	0.079
Gender					
Women	2	0.75	0.65 , 0.88	0.0	0.993
Men	2	0.77	0.58 , 1.01	11.6	0.287
Men and women	6	1.16	0.94 , 1.44	0.0	0.644
Age					
<50	1	0.75	0.61 , 0.92	NA	
≥50	9	0.90	0.79 , 1.04	39.6	0.104
Follow-up					
<10 years	4	1.20	0.89 , 1.61	0.0	0.547
≥10 years	7	0.81	0.71 , 0.91	33.4	0.185
Geographic location					
Europe	6	1.17	0.91 , 1.51	17.6	0.300
America	3	0.76	0.67 , 0.87	0.0	0.937
Australia	1	1.00	0.69 , 1.45	NA	
Number of cases					
<1000	7	1.12	0.90 , 1.38	8.2	0.366
≥1000	3	0.76	0.67 , 0.87	0.0	0.937

Supplementary Table 11 Dose-response meta-analysis for each 50 g/day increase in red meat and risk of type 2 diabetes, stratified by high vs. low intake, gender, age, follow-up, geographic location, number of cases

Dietary factor	No of studies	RR	95% CI	I² (%)	P
Red meat					
High vs. low	18	1.22	1.14 , 1.29	60.3	0.001
Dose-response	16	1.11	1.06 , 1.16	76.0	0.000
Gender					
Men and women	3	1.03	0.96 , 1.10	0.0	0.551
Men	6	1.16	1.05 , 1.28	78.4	0.000
women	7	1.11	1.04 , 1.18	76.3	0.000
Age					
<50	2	1.08	0.98 , 1.19	77.0	0.037
≥50	14	1.12	1.06 , 1.18	77.2	0.000
Follow-up					
<10 years	4	1.05	0.93 , 1.17	65.4	0.034
≥10 years	12	1.14	1.08 , 1.19	72.8	0.000
Geographic location					
Europe	7	1.03	0.98 , 1.09	0.0	0.952
America	6	1.18	1.12 , 1.25	84.1	0.000
Asia	3	1.08	0.87 , 1.34	76.8	0.013
Number of cases					
<1000	8	1.04	0.99 , 1.11	7.5	0.372
≥1000	8	1.14	1.08 , 1.21	84.9	0.000

Supplementary Table 12 Dose-response meta-analysis for each 50 g/day increase in processed meat and risk of type 2 diabetes, stratified by high vs. low intake, gender, age, follow-up, geographic location, number of cases

Dietary factor	No of studies	RR	95% CI	I² (%)	P
Processed meat					
High vs. low	22	1.25	1.15 , 1.35	77.3	0.000
Dose-response	16	1.41	1.24 , 1.60	85.6	0.000
Gender					
Men and women	3	1.12	1.02 , 1.22	0.0	0.627
Men	6	1.67	1.26 , 2.20	83.00.000	
women	7	1.39	1.15 , 1.68	88.00.000	
Age					
<50	2	1.31	0.90 , 1.91	95.0	0.000
≥50	14	1.43	1.24 , 1.65	84.4	0.000
Follow-up					
<10 years	5	1.12	1.06 , 1.19	0.0	0.781
≥10 years	11	1.51	1.31 , 1.74	81.3	0.000
Geographic location					
Europe	8	1.28	1.11 , 1.48	77.9	0.000
America	5	1.72	1.53 , 1.94	45.9	0.116
Asia	3	1.14	1.05 , 1.23	0.0	0.619
Number of cases					
<1000	8	1.14	1.07 , 1.22	0.0	0.929
≥1000	8	1.60	1.32 , 1.94	90.7	0.000

Supplementary Table 13 Dose-response meta-analysis for each 50 g/day increase in fish and risk of type 2 diabetes, stratified by high vs. low intake, gender, age, follow-up, geographic location, number of cases

Dietary factor	No of studies	RR	95% CI	I² (%)	P
Fish					
High vs. low	20	1.08	1.00 , 1.18	76.6	0.000
Dose-response	8	0.99	0.92 , 1.07	61.9	0.010
Gender					
Men and women	2	1.18	0.92 , 1.51	52.1	0.148
Men	3	0.92	0.86 , 0.99	0.0	0.657
women	3	1.00	0.87 , 1.16	76.1	0.015
Age					
<50	1	1.08	0.94 , 1.24	NA	
≥50	7	0.98	0.90 , 1.06	61.3	0.007
Follow-up					
<10 years	4	0.98	0.91 , 1.04	28.4	0.242
≥10 years	4	0.95	0.90 , 1.01	78.5	0.003
Geographic location					
Europe	4	1.08	0.93 , 1.26	71.2	0.016
Asia	4	0.94	0.87 , 0.98	0.0	0.511
Number of cases					
<1000	7	0.99	0.94 , 1.05	59.1	0.023
≥1000	1	0.90	0.83 , 0.98	NA	

Supplementary Table 14 Dose-response meta-analysis for each 50 g/day increase in poultry and risk of type 2 diabetes, stratified by high vs. low intake, gender, age, follow-up, geographic location, number of cases

Dietary factor	No of studies	RR	95% CI	I² (%)	P
Poultry					
High vs. low	12	1.04	1.00 , 1.08	17.7	0.270
Dose-response	8	1.02	0.98 , 1.07	0.0 0.685	
Gender					
Men and women	2	1.05	0.95 , 1.17	0.0	0.615
Men	3	1.05	0.98 , 1.13	0.0	0.625
women	3	0.97	0.90 , 1.05	0.0	0.814
Age					
<50	NA				
≥50	8	1.02	0.98 , 1.07	0.0 0.685	
Follow-up					
<10 years	3	0.98	0.77 , 1.25	0.00.680	
≥10 years	5	1.02	0.98 , 1.07	0.0	0.418
Geographic location					
Europe	4	1.01	0.96 , 1.07	0.0	0.615
USA	2	1.00	0.96 , 1.04	54.4	0.139
Asia	2	1.15	0.74 , 1.80	0.0	0.779
Number of cases					
<1000	4	0.96	0.78 , 1.19	0.00.824	
≥1000	4	1.02	0.98 , 1.08	15.7	0.313

Supplementary Table 15 Dose-response meta-analysis for each 100 g/day increase in milk and risk of type 2 diabetes, stratified by high vs. low intake, gender, age, follow-up, geographic location, number of cases

Dietary factor	No of studies	RR	95% CI	I² (%)	P
Milk					
High vs. low	15	0.98	0.93 , 1.02	27.5	0.153
Dose-response	7	1.01	1.00 , 1.03	5.3	0.386
Gender					
Men and women	5	1.02	1.00 , 1.04	0.0	0.580
Men	1	1.00	0.96 , 1.04	NA	
women	1	0.97	0.92 , 1.02	NA	
Age					
<50NA					
≥50	7	1.01	1.00, 1.03	5.3	0.386
Follow-up					
<10 years	4	0.99	0.97, 1.02	3.2	0.377
≥10 years	3	1.02	1.00 , 1.04	0.0	0.719
Geographic location					
Europe	5	1.02	1.00 , 1.04	0.0	0.580
Asia	2	0.99	0.96 , 1.02	0.0	0.361
Number of cases					
<1000	6	1.00	0.97 , 1.02	0.0	0.500
≥1000	1	1.02	1.00, 1.04	NA	

Supplementary Table 16 Dose-response meta-analysis for each 100 g/day increase in yogurt and risk of type 2 diabetes, stratified by high vs. low intake, gender, age, follow-up, geographic location

Dietary factor	No of studies	RR	95% CI	I^2 (%)	P
Yogurt					
High vs. low	12	0.83	0.77 , 0.89	29.4	0.157
Dose-response	9	0.86	0.81 , 0.92	48.90.048	
Gender					
Men and women	5	0.86	0.79 , 0.94	62.0 0.032	
Men	2	0.92	0.82 , 1.04	10.1	0.292
women	2	0.73	0.60 , 0.89	0.0	0.895
Age					
<50	0	NA			
≥50	9	0.86	0.81 , 0.92	48.90.048	
Follow-up					
<10 years	4	0.87	0.77 , 0.98	63.8	0.040
≥10 years	5	0.86	0.80 , 0.93	45.60.119	
Geographic location					
Europe	4	0.87	0.78 , 0.98	71.3 0.015	
USA	2	0.86	0.77 , 0.97	53.9	0.141
Asia	3	0.86	0.77 , 0.95	33.3	0.223
Number of cases					
<1000	6	0.88	0.79 , 0.97	62.5	0.020
≥1000	3	0.86	0.79 , 0.93	9.3	0.332

Supplementary Table 17 Dose-response meta-analysis for each 50g/day increase in soy and risk of type 2 diabetes, stratified by high vs. low intake, gender, age, follow-up, geographic location, number of cases

Dietary factor	No of studies	RR	95% CI	I^2 (%)	P
Soy					
High vs. low	24	1.00	0.90 , 1.10	82.0	0.000
Dose-response	19	1.15	0.97 , 1.37	85.2	0.000
Gender					
Men and women	4	0.83	0.65 , 1.06	51.4	0.104
Men	8	1.35	1.00 , 1.82	81.6	0.000
women	7	1.36	0.93 , 2.01	91.3	0.000
Age					
<50	1	0.98	0.89 , 1.08	NA	
≥50	18	1.19	0.97, 1.47	86.0	0.000
Follow-up					
<10 years	9	0.81	0.68 , 0.96	80.0	0.000
≥10 years	10	1.90	1.38 , 2.60	79.5	0.000
Geographic location					
Europe	5	0.96	0.85 , 1.09	38.0	0.168
USA	8	2.27	1.77 , 2.90	27.1 0.212	
Asia	6	0.78	0.61 , 1.00	84.2	0.000
Number of cases					
<1000	12	1.02	0.87 , 1.18	72.0	0.000
≥1000	7	1.46	0.82 , 2.57	92.5	0.000

Supplementary Table18 Dose-response meta-analysis for each 50g/day increase in egg and risk of type 2 diabetes, stratified by high vs. low intake, gender, age, follow-up, geographic location, number of cases

Dietary factor	No of studies	RR	95% CI	I^2 (%)	P
Egg					
High vs. low	19	1.10	1.03 , 1.16	54.8	0.002
Dose-response	19	1.01	0.99 , 1.03	70.8	0.000
Gender					
Men and women	8	1.02	1.01 , 1.03	0.2	0.427
Men	5	0.99	0.94 , 1.04	82.5	0.000
women	4	1.02	0.97 , 1.06	82.6	0.000
Age					
<50	2	0.96	0.87 , 1.05	31.3	0.228
≥50	15	1.01	1.00 , 1.03	72.2	0.000
Follow-up					
<10 years	6	0.99	0.96 , 1.02	51.9	0.042
≥10 years	11	1.03	1.01 , 1.05	70.0	0.000
Geographic location					
Europe	8	1.01	0.99 , 1.03	42.5	0.095
USA	7	1.04	1.02 , 1.07	69.00.004	
Asia	2	0.96	0.90 , 1.02	65.7	0.033
Number of cases					
<1000	11	1.00	0.97 , 1.03	75.4	0.000
≥1000	6	1.02	1.00 , 1.04	56.4	0.043

Supplementary Table 19 Dose-response meta-analysis for each 30g/day increase in cheese and risk of type 2 diabetes, stratified by high vs. low intake, gender, age, follow-up, geographic location, number of cases

Dietary factor	No of studies	RR	95% CI	I^2 (%)	P
Cheese					
High vs. low	15	0.94	0.89 , 1.00	1.9	0.430
Dose-response	11	0.97	0.93 , 1.03	13.9	0.312
Gender					
Men and women	6	0.98	0.94 , 1.03	5.2	0.383
Men	3	0.99	0.88 , 1.11	0.0	0.789
women	2	0.78	0.64 , 0.96	25.9	0.245
Age					
<50	0	NA			
≥50	11	0.97	0.93 , 1.03	13.9	0.312
Follow-up					
<10 years	4	1.05	0.89 , 1.25	0.0	0.397
≥10 years	7	0.97	0.92 , 1.01	22.1	0.261
Geographic location					
Europe	6	0.99	0.94 , 1.03	0.0	0.438
USA	3	0.83	0.71 , 0.96	0.00.495	
Asia	2	1.13	0.47 , 2.72	0.0	0.411
Number of cases					
<1000	8	1.03	0.94 , 1.13	0.0	0.671
≥1000	3	0.96	0.91 , 1.00	56.7	0.099

Supplementary figures:

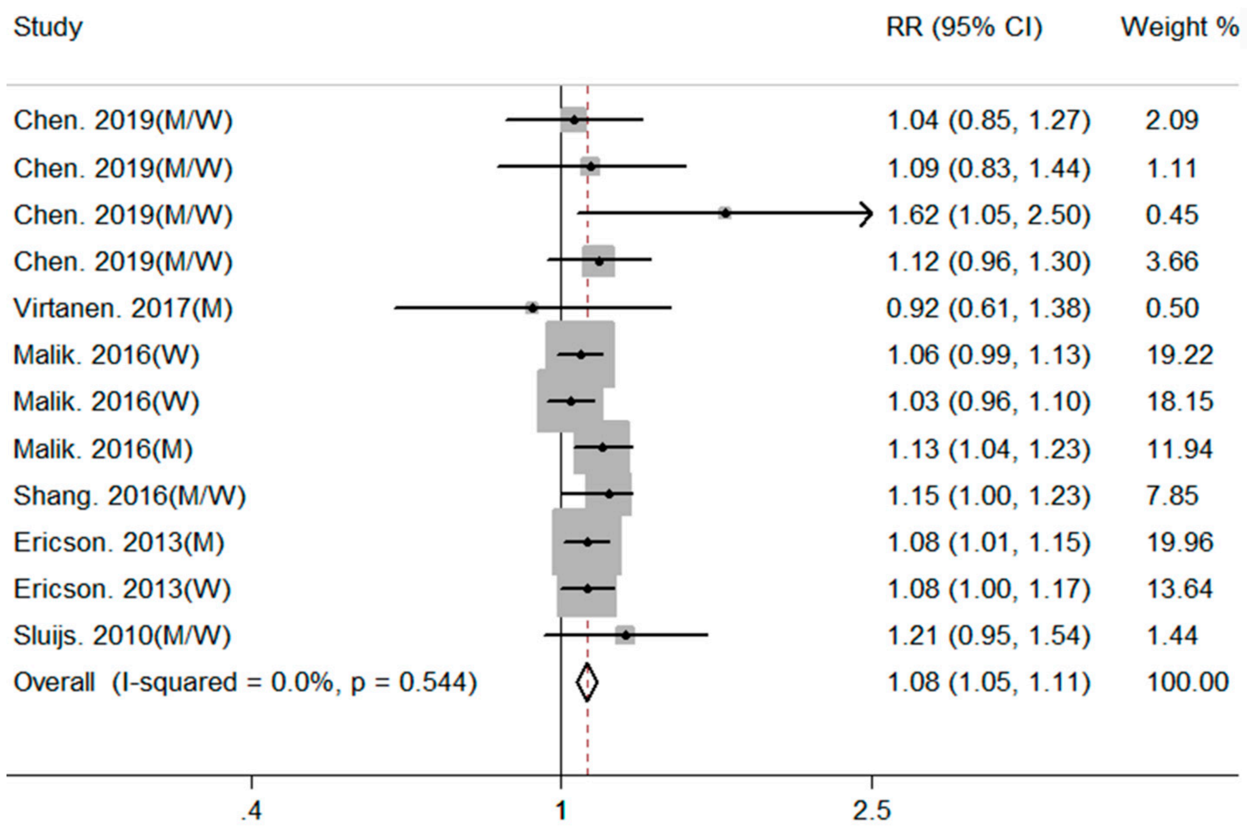

Supplementaryfigure 1. Prospective associationsof dietary total protein intake withincident type 2 diabetes for per5% of energy increase

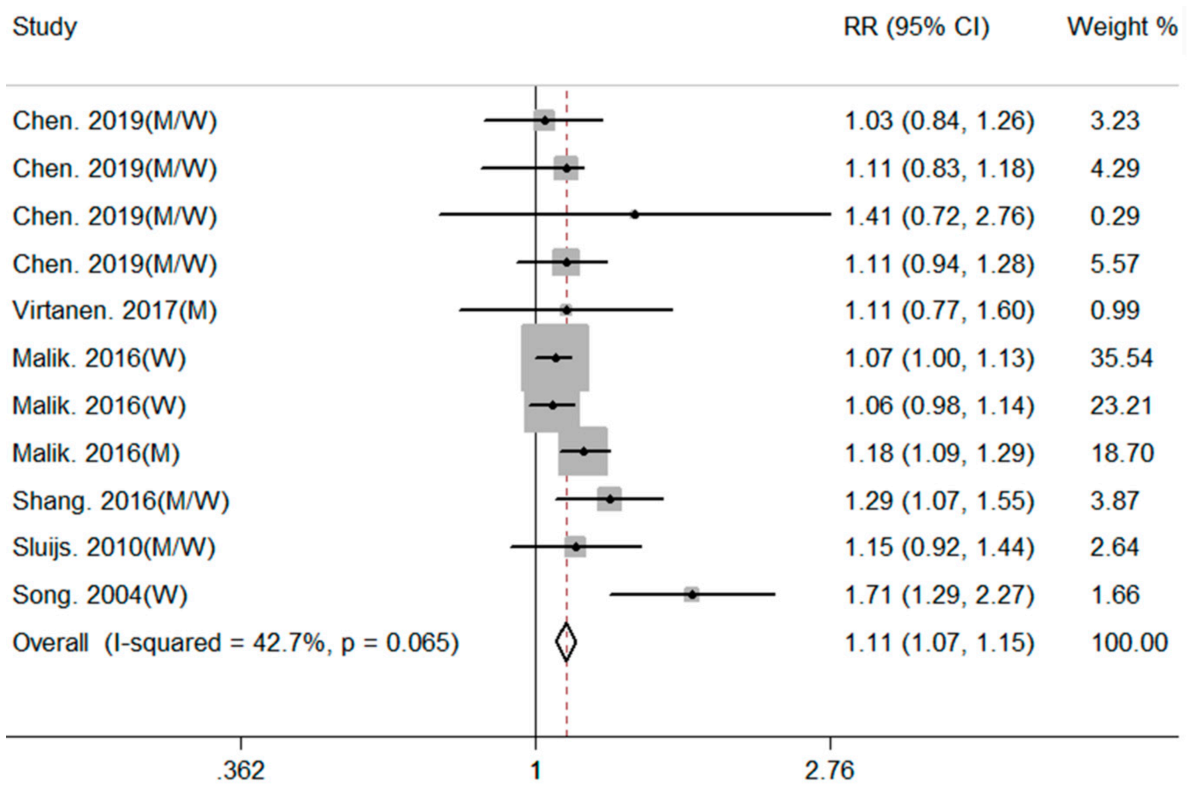

Supplementaryfigure 2. Prospective associationsof dietary animal protein intake withincident type 2 diabetes for per5% of energy increase

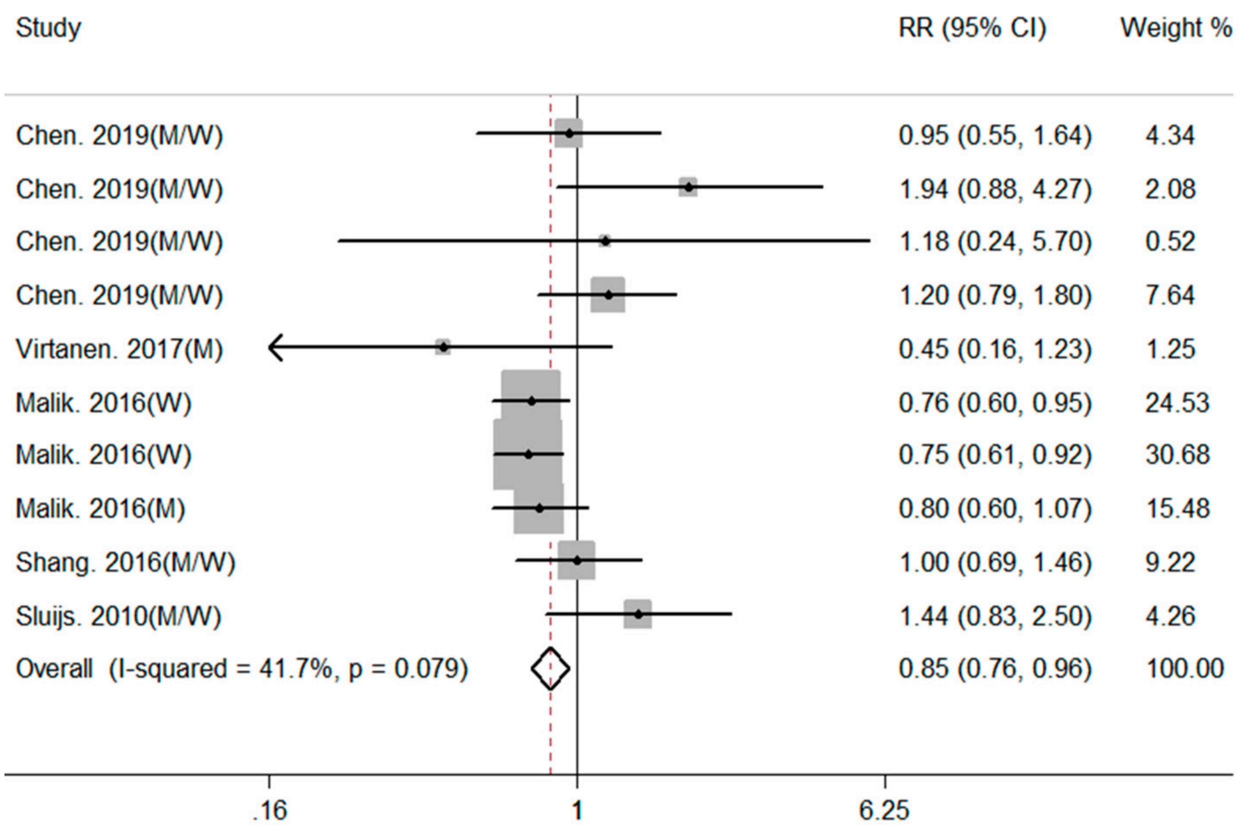

Supplementaryfigure 3. Prospective associationsof dietary plant protein intake withincident type 2 diabetes for per5% of energy increase

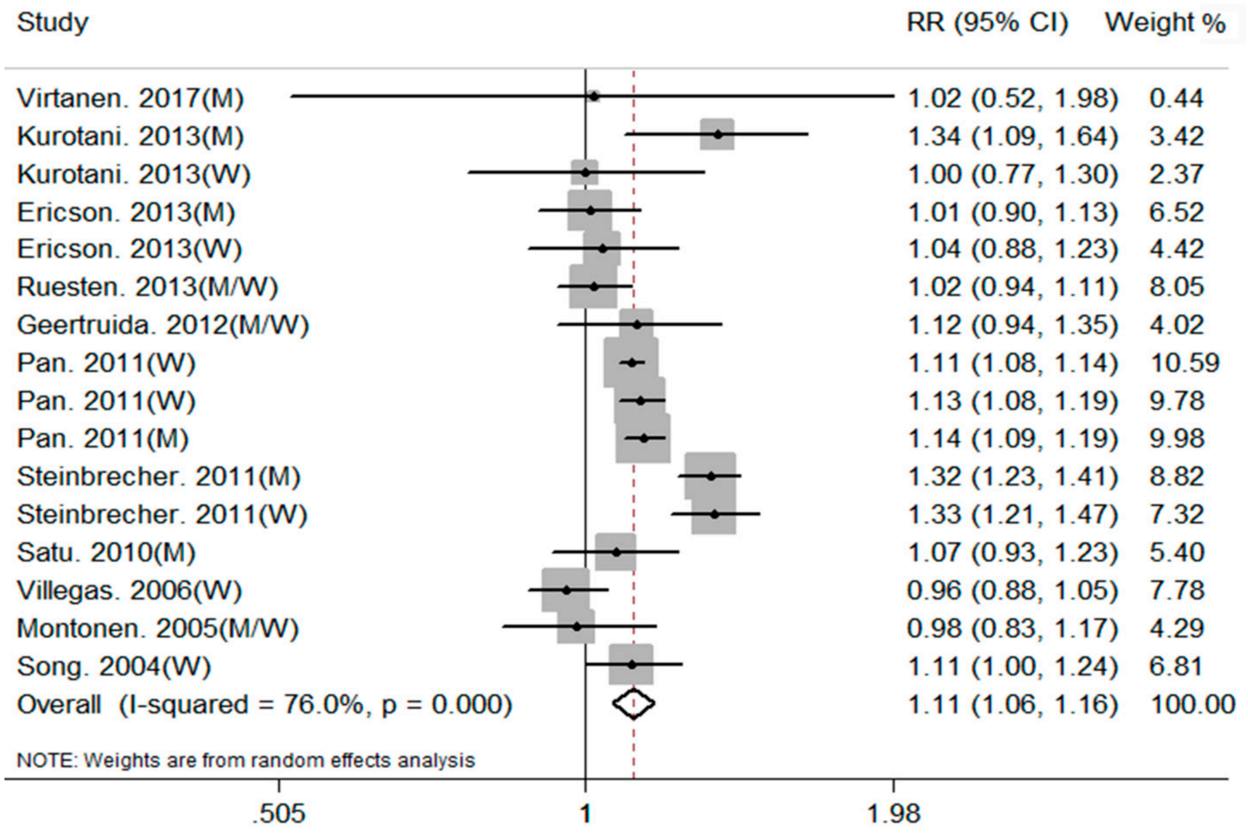

Supplementaryfigure 4. Prospective associationsof dietary red meat intake withincident type 2 diabetes for per50g/day increase

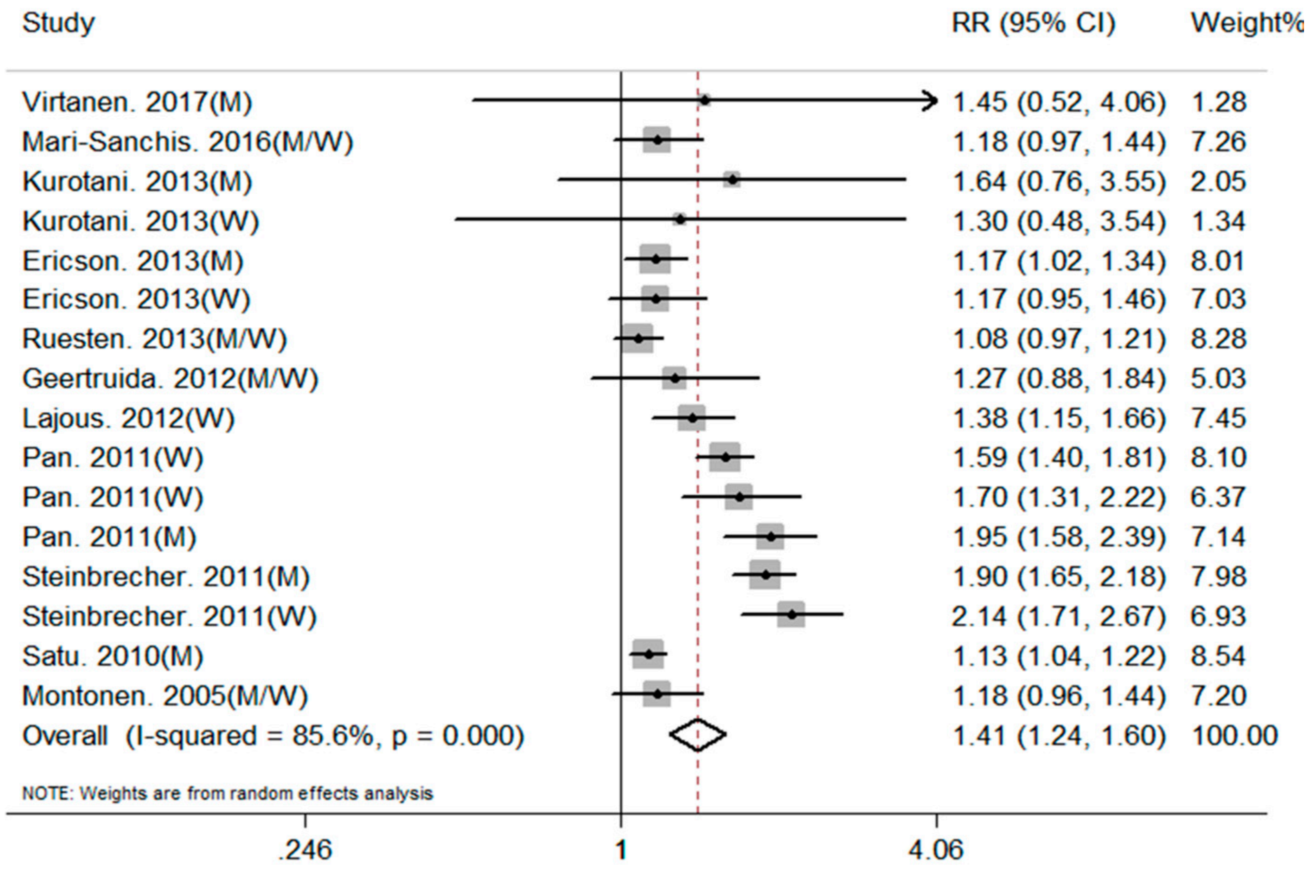

Supplementaryfigure 5. Prospective associationsof dietary processed meat intake withincident type 2 diabetes forper 50g/day increase

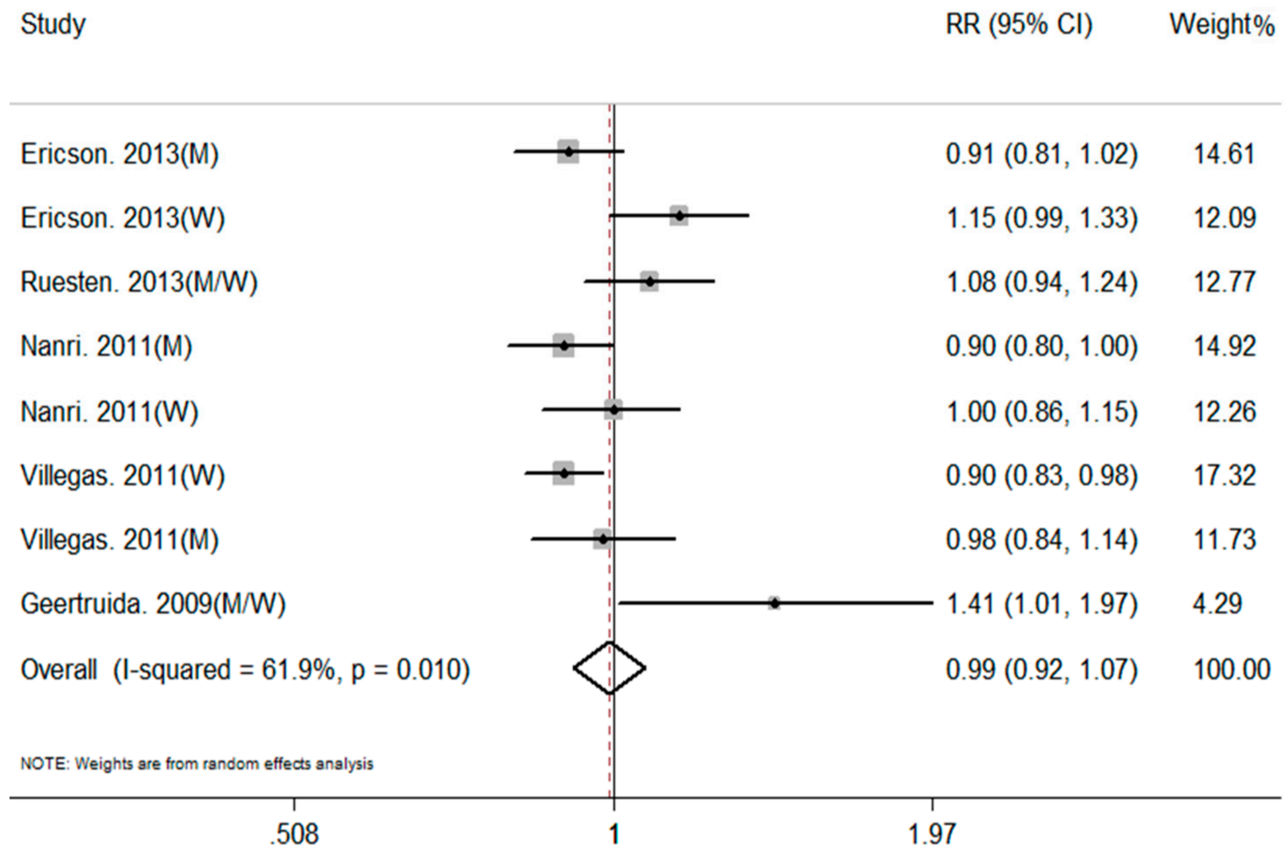

Supplementaryfigure 6. Prospective associationsof dietary fish intake withincident type 2 diabetes forper 50g/day increase

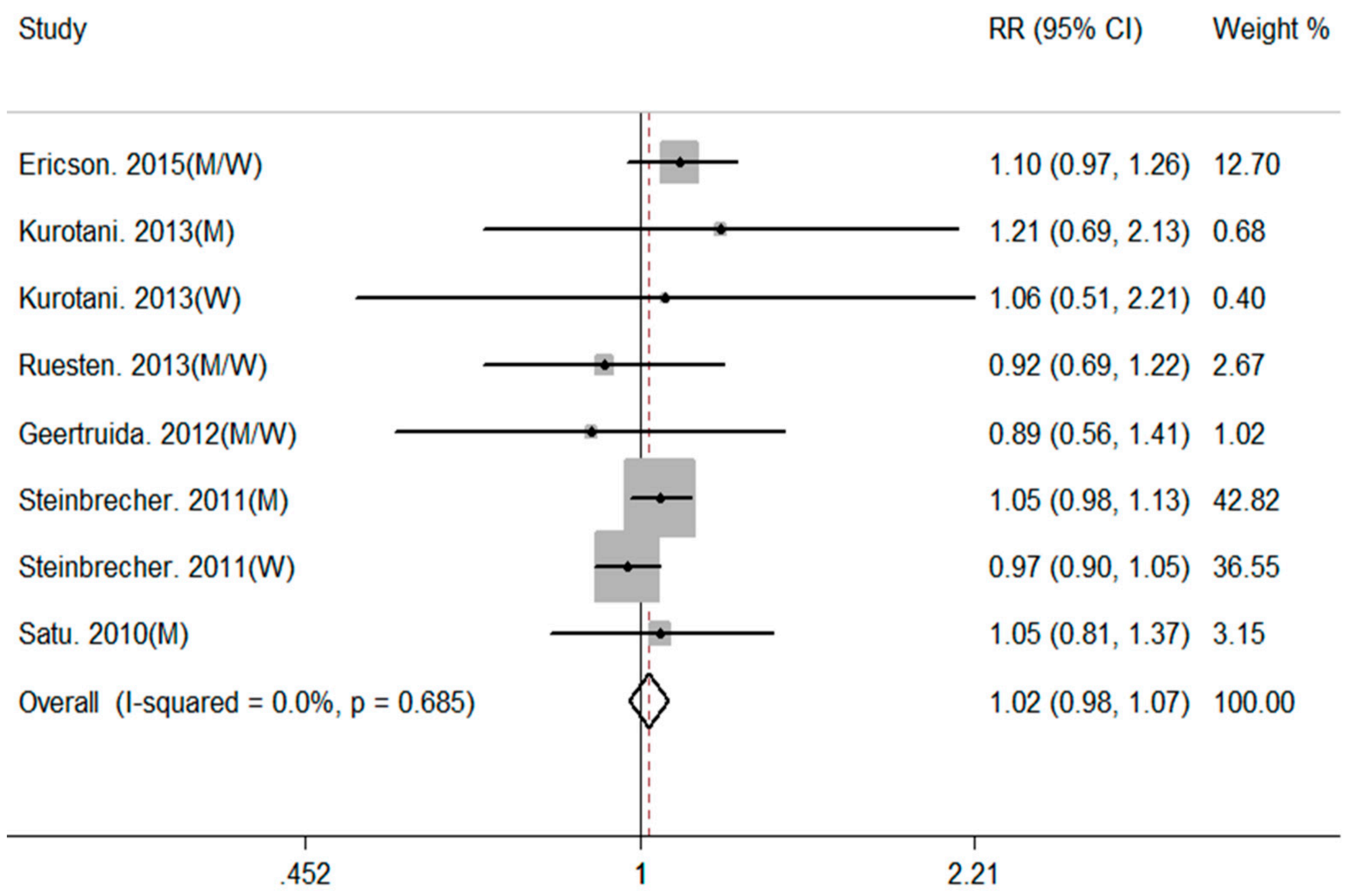

Supplementaryfigure 7. Prospective associationsof dietary poultry intake withincident type 2 diabetes forper 50g/day increase

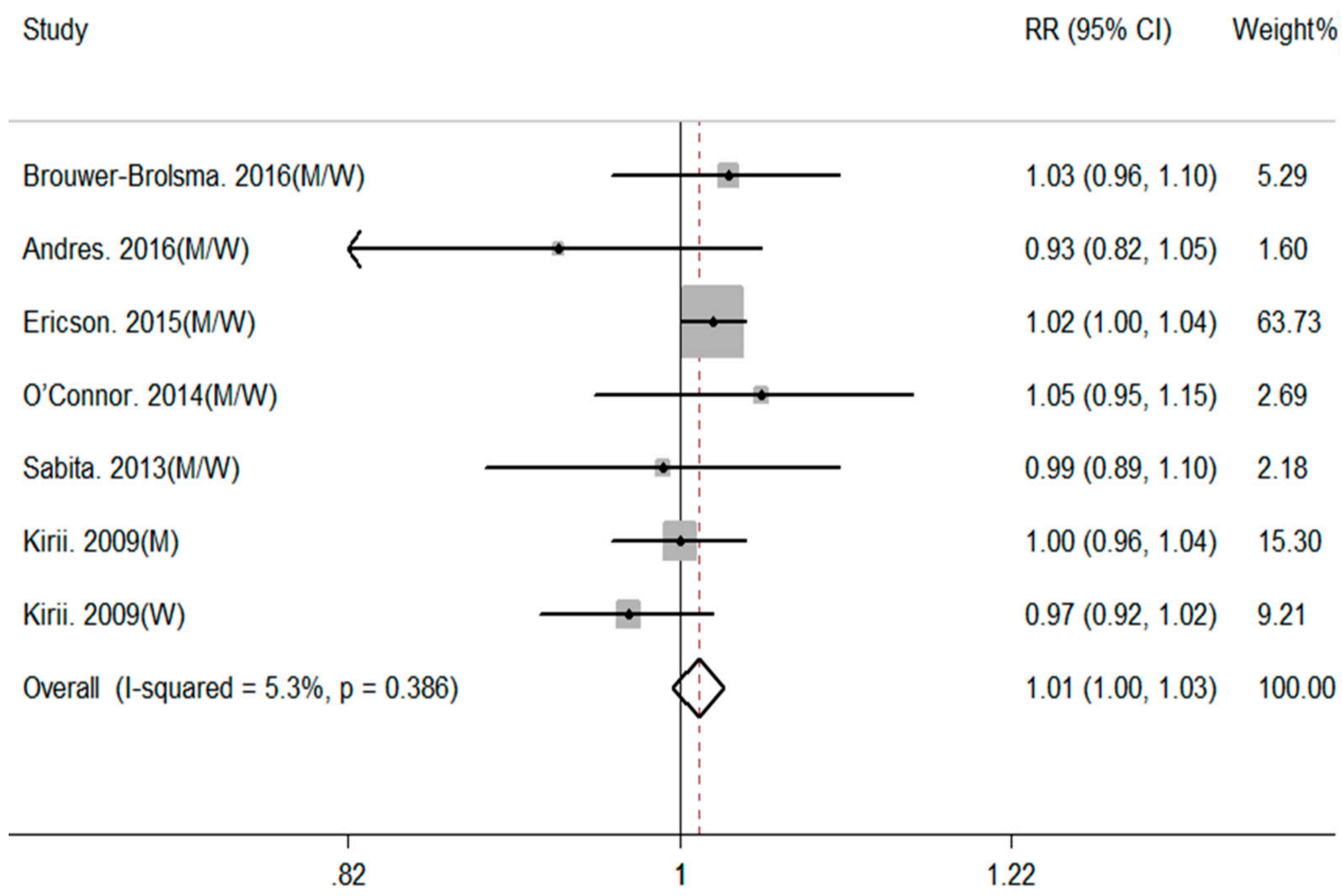

Supplementaryfigure8. Prospective associationsof dietary milk intake withincident type 2 diabetes forper 100g/day increase

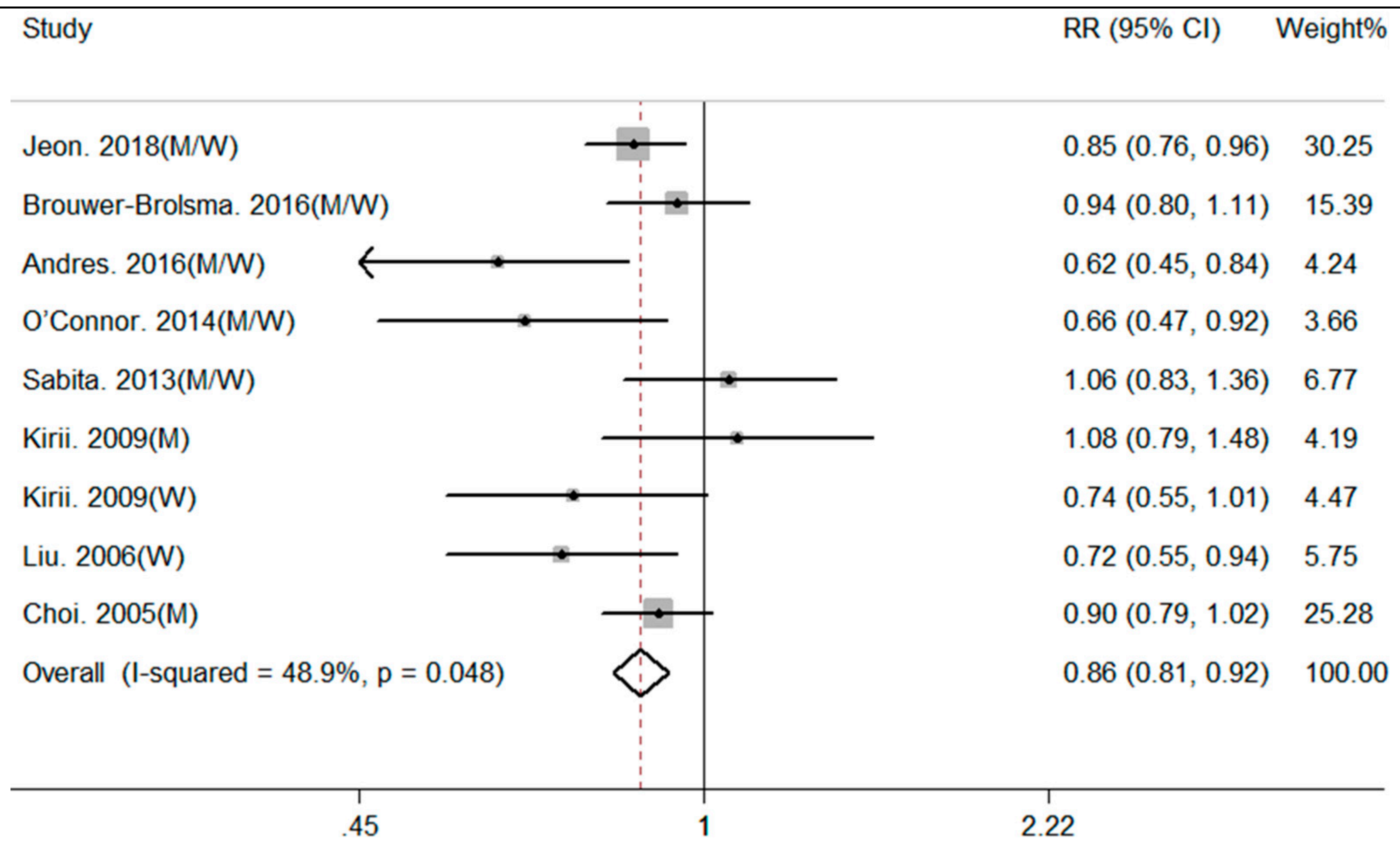

Supplementaryfigure 9. Prospective associationsof dietary yogurt intake withincident type 2 diabetes forper 100g/day increase

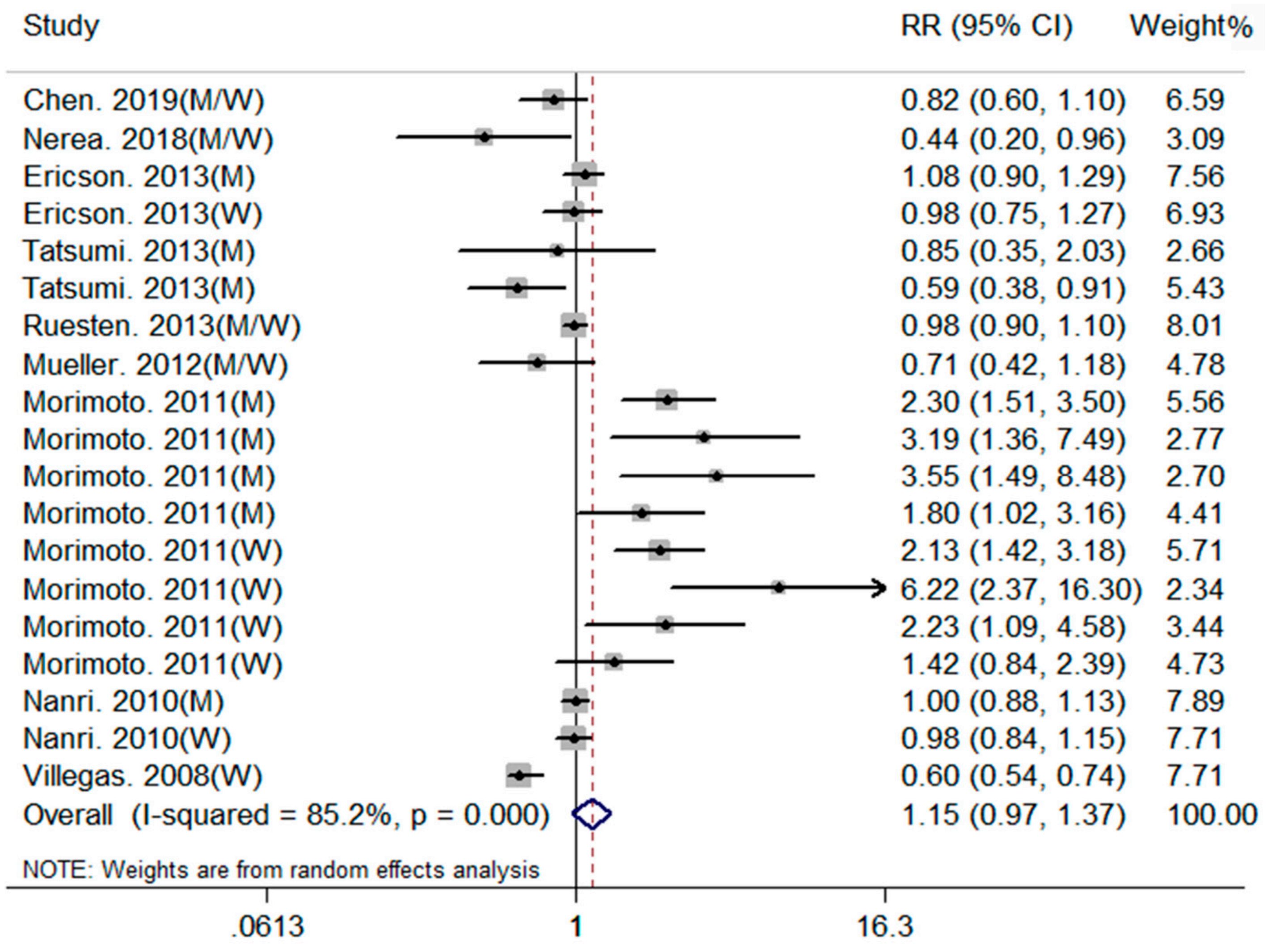

Supplementaryfigure 10. Prospective associationsof dietary soy intake withincident type 2 diabetes forper 50g/day increase

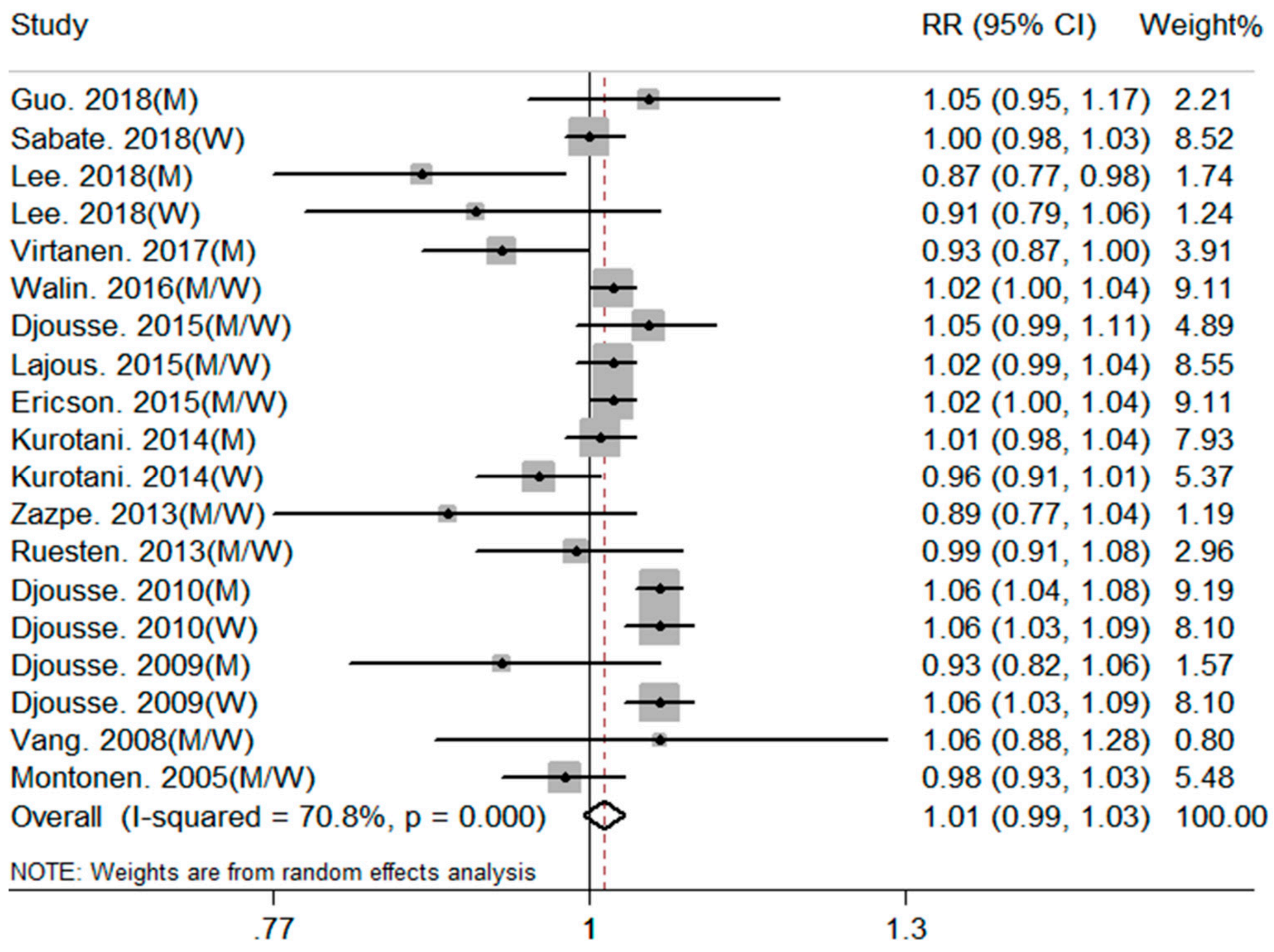

Supplementaryfigure 11. Prospective associationsof dietary egg withincident type 2 diabetes forper50g/day increase

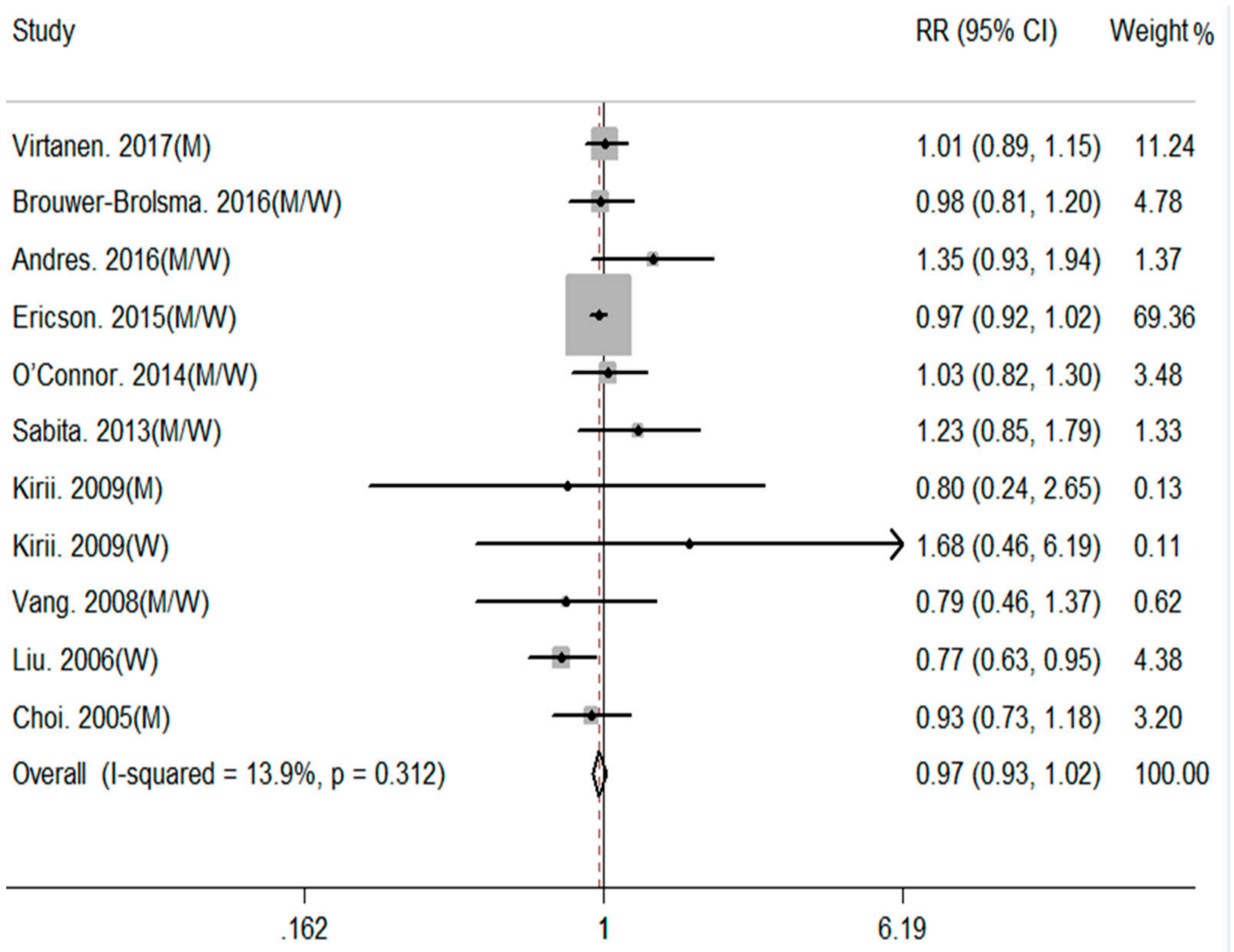

Supplementaryfigure 12. Prospective associationsof dietary cheese withincident type 2 diabetes forper 30g/day increase

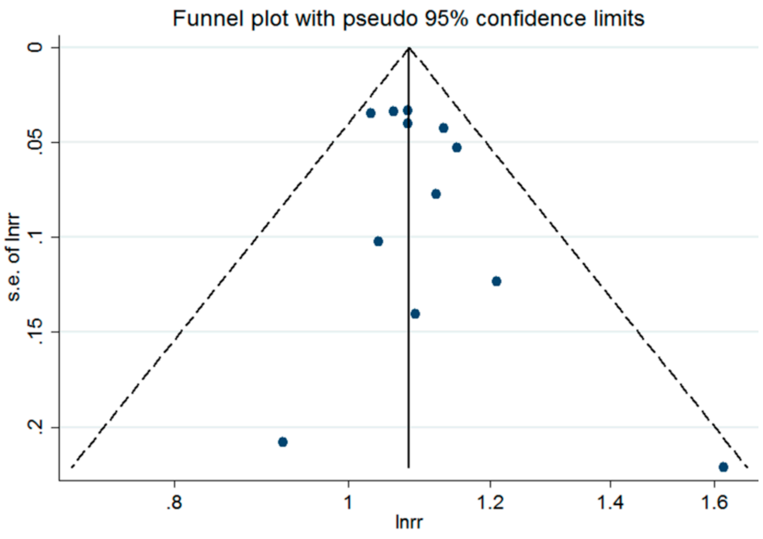

Supplementary Figure 13: Funnel plots of dietary total protein-T2D associations(dose-response meta-analysis) SE = Standard error

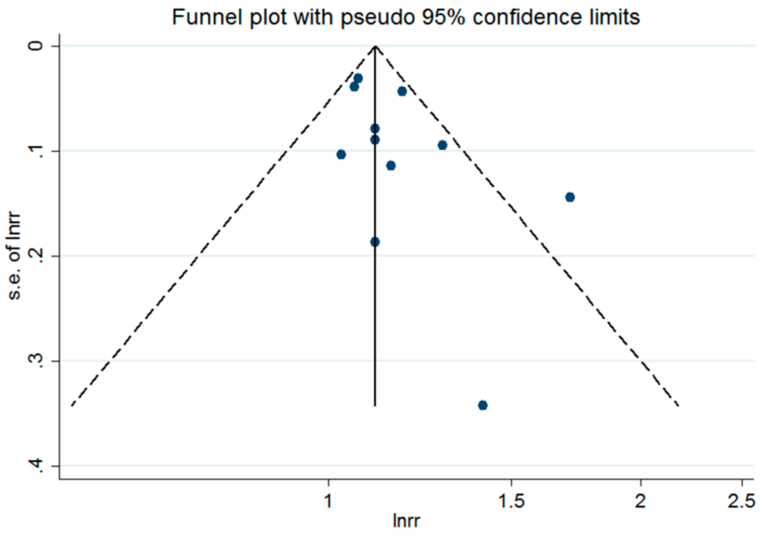

Supplementary Figure 14: Funnel plots of dietary animal protein-T2D associations(dose-response meta-analysis) SE = Standard error

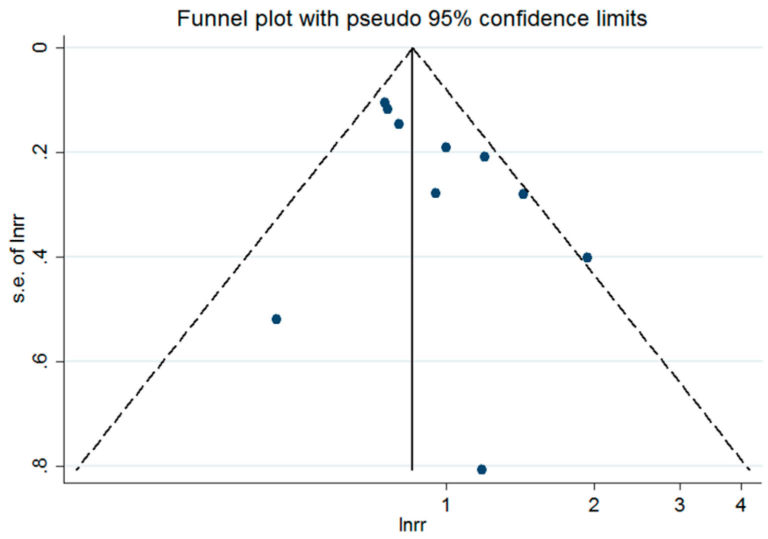

Supplementary Figure 15: Funnel plots of dietary plant protein-T2D associations(dose-response meta-analysis) SE = Standard error

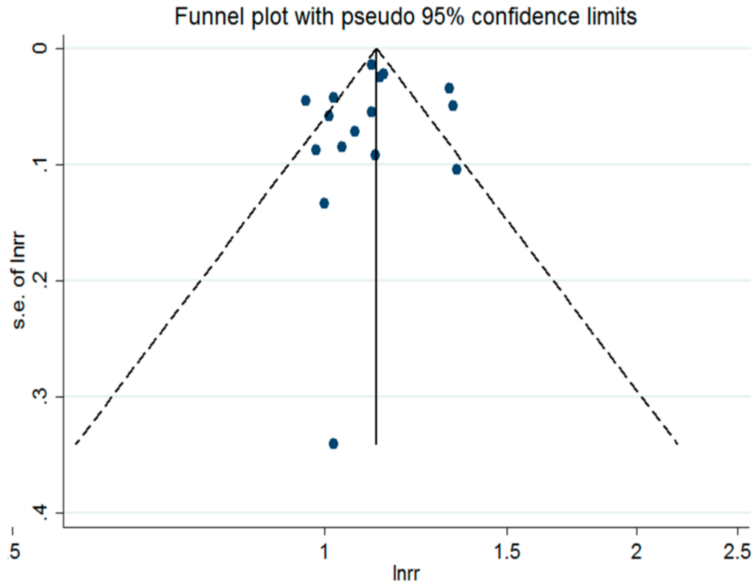

Supplementary Figure 16: Funnel plots of dietary red meat-T2D associations(dose-response meta-analysis) SE = Standard error

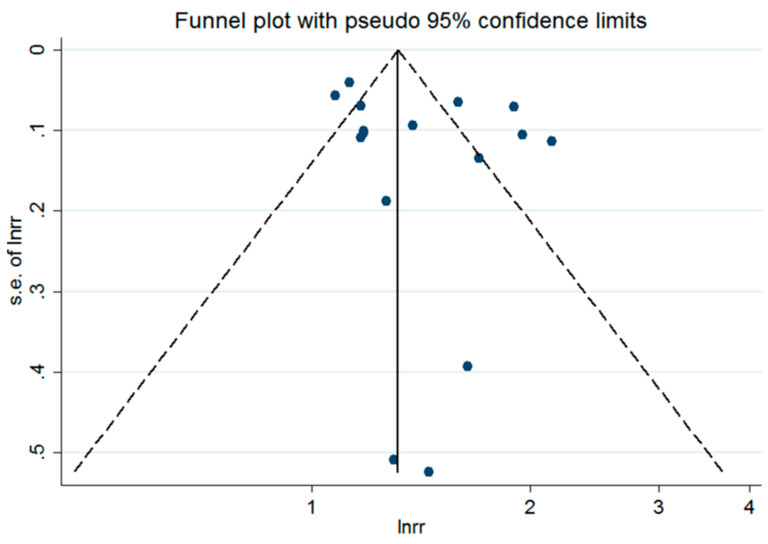

Supplementary Figure 17: Funnel plots of dietary processed meat-T2D associations(dose-response meta-analysis) SE = Standard error

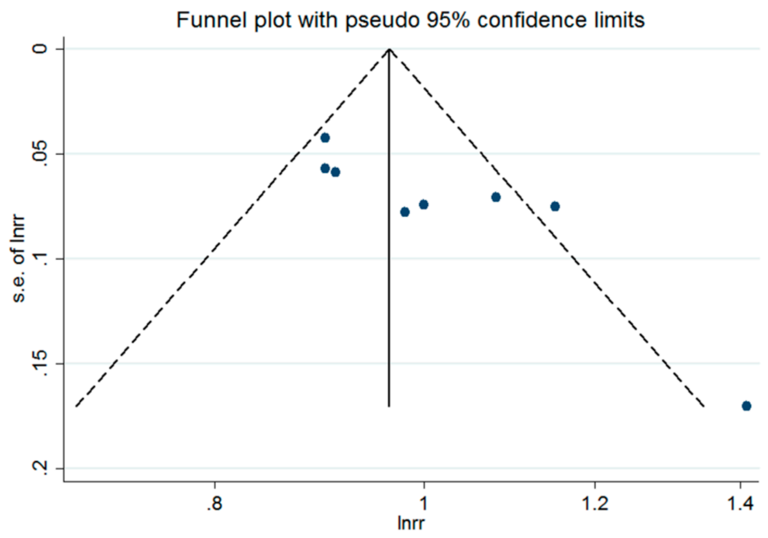

Supplementary Figure 18: Funnel plots of dietary fish-T2D associations(dose-response meta-analysis) SE = Standard error

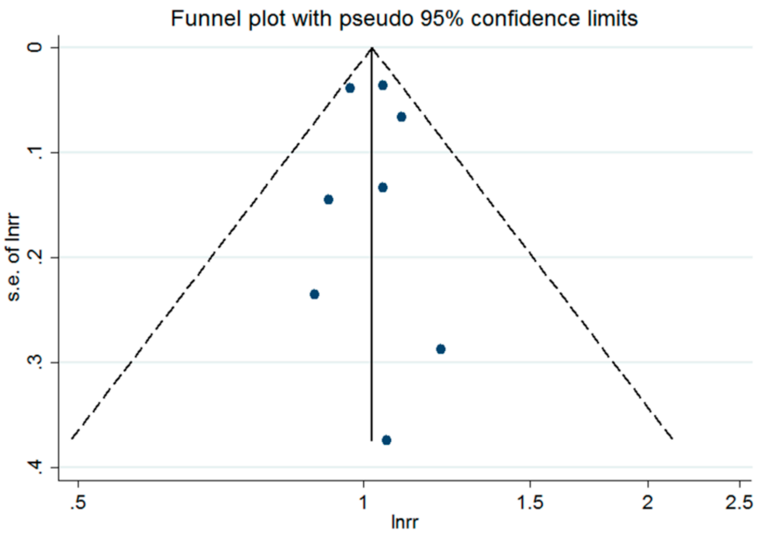

Supplementary Figure 19: Funnel plots of dietary poultry-T2D associations(dose-response meta-analysis) SE = Standard error

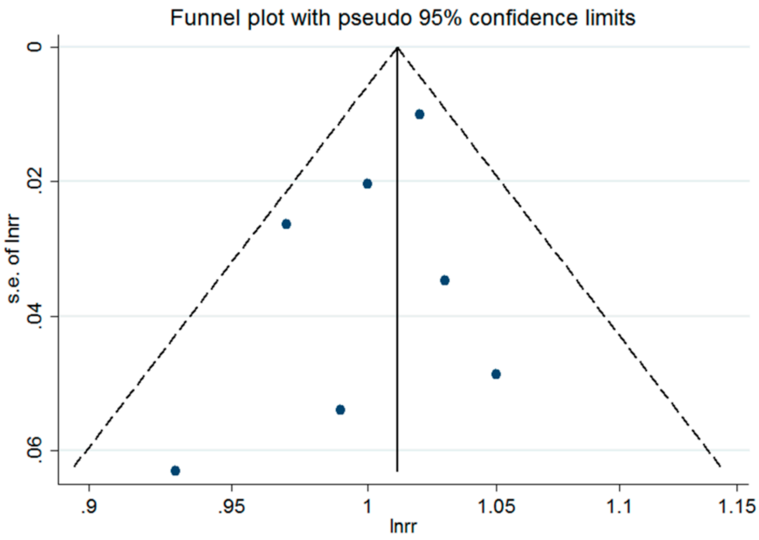

Supplementary Figure 20: Funnel plots of dietary milk-T2D associations(dose-response meta-analysis) SE = Standard error

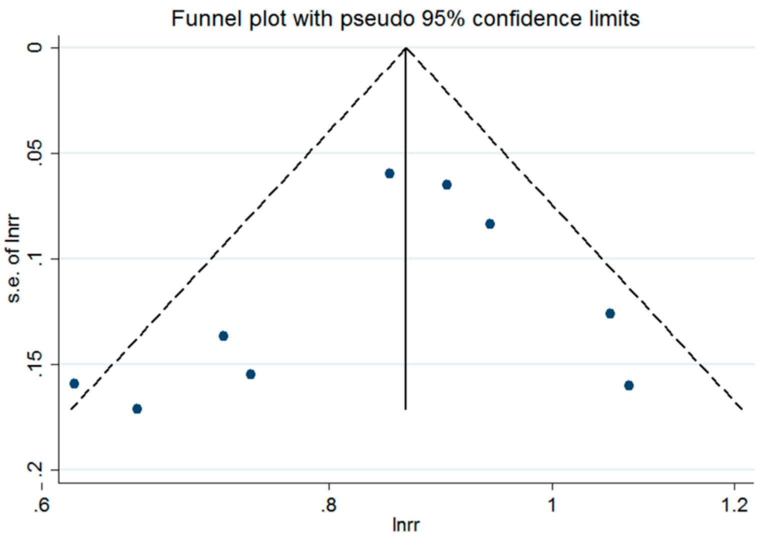

Supplementary Figure 21: Funnel plots of dietary yogurt-T2D associations(dose-response meta-analysis) SE = Standard error

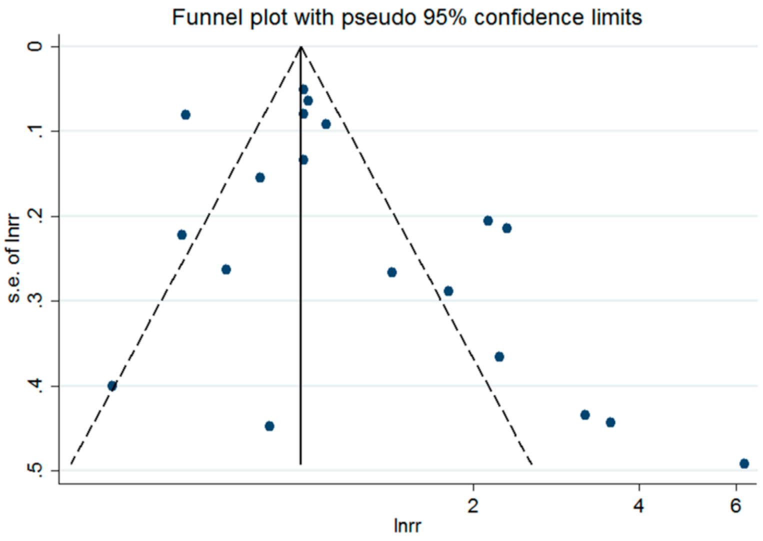

Supplementary Figure 22: Funnel plots of dietary soy-T2D associations(dose-response meta-analysis) SE = Standard error

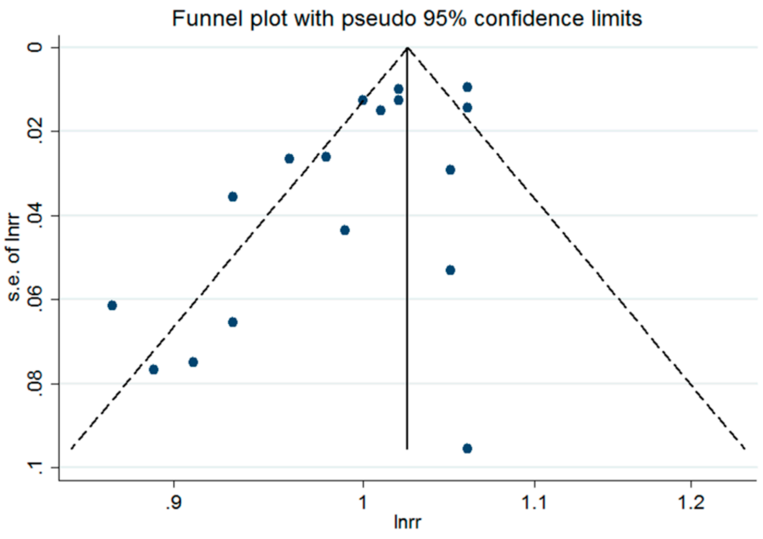

Supplementary Figure 23: Funnel plots of dietary egg-T2D associations(dose-response meta-analysis) SE = Standard error

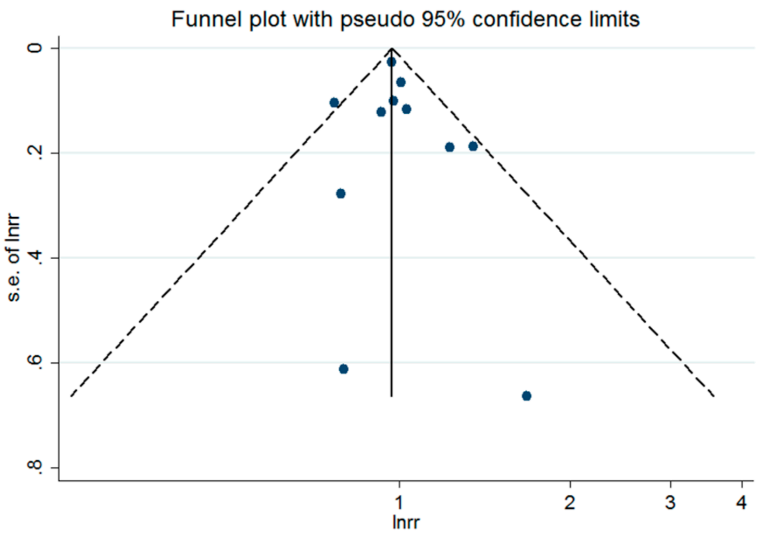

Supplementary Figure 24: Funnel plots of dietary cheese-T2D associations(dose-response meta-analysis) SE = Standard error

SupplementaryReferences

1. Chen, Z.; Franco, O.H.; Lamballais, S.; Ikram, M.A.; Schoufour, J.D.; Muka, T.; Voortman, T. Associations of specific dietary protein with longitudinal insulin resistance, prediabetes and type 2 diabetes: The Rotterdam Study. *Clinical nutrition* **2019**, 10.1016/j.clnu.2019.01.021, doi:10.1016/j.clnu.2019.01.021.
2. Talaei, M.; Pan, A.; Yuan, J.M.; Koh, W.P. Dairy intake and risk of type 2 diabetes. *Clinical nutrition* **2018**, *37*, 712-718, doi:10.1016/j.clnu.2017.02.022.
3. Jeon, J.; Jang, J.; Park, K. Effects of Consuming Calcium-Rich Foods on the Incidence of Type 2 Diabetes Mellitus. *Nutrients* **2018**, *11*, doi:10.3390/nu11010031.
4. Talaei, M.; Wang, Y.L.; Yuan, J.M.; Pan, A.; Koh, W.P. Meat, Dietary Heme Iron, and Risk of Type 2 Diabetes Mellitus: The Singapore Chinese Health Study. *American journal of epidemiology* **2017**, *186*, 824-833, doi:10.1093/aje/kwx156.
5. Becerra-Tomas, N.; Diaz-Lopez, A.; Rosique-Esteban, N.; Ros, E.; Buil-Cosiales, P.; Corella, D.; Estruch, R.; Fito, M.; Serra-Majem, L.; Aros, F., et al. Legume consumption is inversely associated with type 2 diabetes incidence in adults: A prospective assessment from the PREDIMED study. *Clinical nutrition* **2018**, *37*, 906-913, doi:10.1016/j.clnu.2017.03.015.
6. Lee, J.; Kim, J. Egg consumption is associated with a lower risk of type 2

diabetes in middle-aged and older men. *Nutrition research and practice* **2018**, *12*, 396-405, doi:10.4162/nrp.2018.12.5.396.

7. Son, J.; Lee, Y.; Park, K. Effects of processed red meat consumption on the risk of type 2 diabetes and cardiovascular diseases among Korean adults: the Korean Genome and Epidemiology Study. *European journal of nutrition* **2018**, 10.1007/s00394-018-1799-6, doi:10.1007/s00394-018-1799-6.
8. Sabaté, J.; Burkholder-Cooley, N.M.; Segovia-Siapco, G.; Oda, K.; Wells, B.; Orlich, M.J.; Fraser, G.E. Unscrambling the relations of egg and meat consumption with type 2 diabetes risk. *American Journal of Clinical Nutrition* **2018**, *108*, 1121-1128, doi:10.1093/ajcn/nqy213.
9. Isanejad, M.; LaCroix, A.Z.; Thomson, C.A.; Tinker, L.; Larson, J.C.; Qi, Q.; Qi, L.; Cooper-DeHoff, R.M.; Phillips, L.S.; Prentice, R.L., et al. Branched-chain amino acid, meat intake and risk of type 2 diabetes in the Women's Health Initiative. *The British journal of nutrition* **2017**, *117*, 1523-1530, doi:10.1017/S0007114517001568.
10. Virtanen, H.E.K.; Koskinen, T.T.; Voutilainen, S.; Mursu, J.; Tuomainen, T.P.; Kokko, P.; Virtanen, J.K. Intake of different dietary proteins and risk of type 2 diabetes in men: the Kuopio Ischaemic Heart Disease Risk Factor Study. *The British journal of nutrition* **2017**, *117*, 882-893, doi:10.1017/S0007114517000745.
11. Wallin, A.; Di Giuseppe, D.; Orsini, N.; Akesson, A.; Forouhi, N.G.; Wolk, A.

Fish consumption and frying of fish in relation to type 2 diabetes incidence: a prospective cohort study of Swedish men. *European journal of nutrition* **2017**, *56*, 843-852, doi:10.1007/s00394-015-1132-6.

12. Malik, V.S.; Li, Y.; Tobias, D.K.; Pan, A.; Hu, F.B. Dietary Protein Intake and Risk of Type 2 Diabetes in US Men and Women. *American journal of epidemiology* **2016**, *183*, 715-28, doi:10.1093/aje/kwv268.
13. Mari-Sanchis, A.; Gea, A.; Basterra-Gortari, F.J.; Martinez-Gonzalez, M.A.; Beunza, J.J.; Bes-Rastrollo, M. Meat Consumption and Risk of Developing Type 2 Diabetes in the SUN Project: A Highly Educated Middle-Class Population. *PloS one* **2016**, *11*, e0157990, doi:10.1371/journal.pone.0157990.
14. Brouwer-Brolsma, E.M.; van Woudenbergh, G.J.; Oude Elferink, S.J.; Singh-Povel, C.M.; Hofman, A.; Dehghan, A.; Franco, O.H.; Feskens, E.J. Intake of different types of dairy and its prospective association with risk of type 2 diabetes: The Rotterdam Study. *Nutrition, metabolism, and cardiovascular diseases : NMCD* **2016**, *26*, 987-995, doi:10.1016/j.numecd.2016.08.003.
15. Shang, X.; Scott, D.; Hodge, A.M.; English, D.R.; Giles, G.G.; Ebeling, P.R.; Sanders, K.M. Dietary protein intake and risk of type 2 diabetes: results from the Melbourne Collaborative Cohort Study and a meta-analysis of prospective studies. *The American journal of clinical nutrition* **2016**, *104*, 1352-1365, doi:10.3945/ajcn.116.140954.

16. Diaz-Lopez, A.; Bullo, M.; Martinez-Gonzalez, M.A.; Corella, D.; Estruch, R.; Fito, M.; Gomez-Gracia, E.; Fiol, M.; Garcia de la Corte, F.J.; Ros, E., et al. Dairy product consumption and risk of type 2 diabetes in an elderly Spanish Mediterranean population at high cardiovascular risk. *European journal of nutrition* **2016**, *55*, 349-60, doi:10.1007/s00394-015-0855-8.

17. Ding, M.; Pan, A.; Manson, J.E.; Willett, W.C.; Malik, V.; Rosner, B.; Giovannucci, E.; Hu, F.B.; Sun, Q. Consumption of soy foods and isoflavones and risk of type 2 diabetes: a pooled analysis of three US cohorts. *European journal of clinical nutrition* **2016**, *70*, 1381-1387, doi:10.1038/ejcn.2016.117.

18. O'Connor, L.M.; Lentjes, M.A.; Luben, R.N.; Khaw, K.T.; Wareham, N.J.; Forouhi, N.G. Dietary dairy product intake and incident type 2 diabetes: a prospective study using dietary data from a 7-day food diary. *Diabetologia* **2014**, *57*, 909-17, doi:10.1007/s00125-014-3176-1.

19. Zong, G.; Sun, Q.; Yu, D.; Zhu, J.; Sun, L.; Ye, X.; Li, H.; Jin, Q.; Zheng, H.; Hu, F.B., et al. Dairy consumption, type 2 diabetes, and changes in cardiometabolic traits: a prospective cohort study of middle-aged and older Chinese in Beijing and Shanghai. *Diabetes care* **2014**, *37*, 56-63, doi:10.2337/dc13-0975.

20. Soedamah-Muthu, S.S.; Masset, G.; Verberne, L.; Geleijnse, J.M.; Brunner, E.J. Consumption of dairy products and associations with incident diabetes, CHD and mortality in the Whitehall II study. *The British journal of nutrition*

2013, *109*, 718-26, doi:10.1017/S0007114512001845.

21. Struijk, E.A.; Heraclides, A.; Witte, D.R.; Soedamah-Muthu, S.S.; Geleijnse, J.M.; Toft, U.; Lau, C.J. Dairy product intake in relation to glucose regulation indices and risk of type 2 diabetes. *Nutrition, metabolism, and cardiovascular diseases : NMCD* **2013**, *23*, 822-8, doi:10.1016/j.numecd.2012.05.011.
22. Kurotani, K.; Nanri, A.; Goto, A.; Mizoue, T.; Noda, M.; Oba, S.; Kato, M.; Matsushita, Y.; Inoue, M.; Tsugane, S., et al. Red meat consumption is associated with the risk of type 2 diabetes in men but not in women: a Japan Public Health Center-based Prospective Study. *The British journal of nutrition* **2013**, *110*, 1910-8, doi:10.1017/S0007114513001128.
23. Ericson, U.; Sonestedt, E.; Gullberg, B.; Hellstrand, S.; Hindy, G.; Wirfalt, E.; Orho-Melander, M. High intakes of protein and processed meat associate with increased incidence of type 2 diabetes. *The British journal of nutrition* **2013**, *109*, 1143-53, doi:10.1017/S0007114512003017.
24. Tatsumi, Y.; Morimoto, A.; Deura, K.; Mizuno, S.; Ohno, Y.; Watanabe, S. Effects of soybean product intake on fasting and postload hyperglycemia and type 2 diabetes in Japanese men with high body mass index: The Saku Study. *Journal of diabetes investigation* **2013**, *4*, 626-33, doi:10.1111/jdi.12100.
25. Lajous, M.; Tondeur, L.; Fagherazzi, G.; de Lauzon-Guillain, B.; Boutron-Ruaualt, M.C.; Clavel-Chapelon, F. Processed and unprocessed red meat consumption and incident type 2 diabetes among French women.

Diabetes care **2012**, *35*, 128-30, doi:10.2337/dc11-1518.

26. Fretts, A.M.; Howard, B.V.; McKnight, B.; Duncan, G.E.; Beresford, S.A.; Mete, M.; Eilat-Adar, S.; Zhang, Y.; Siscovick, D.S. Associations of processed meat and unprocessed red meat intake with incident diabetes: the Strong Heart Family Study. *The American journal of clinical nutrition* **2012**, *95*, 752-8, doi:10.3945/ajcn.111.029942.
27. van Woudenbergh, G.J.; Kuijsten, A.; Tigcheler, B.; Sijbrands, E.J.; van Rooij, F.J.; Hofman, A.; Witteman, J.C.; Feskens, E.J. Meat consumption and its association with C-reactive protein and incident type 2 diabetes: the Rotterdam Study. *Diabetes care* **2012**, *35*, 1499-505, doi:10.2337/dc11-1899.
28. Mueller, N.T.; Odegaard, A.O.; Gross, M.D.; Koh, W.P.; Yu, M.C.; Yuan, J.M.; Pereira, M.A. Soy intake and risk of type 2 diabetes in Chinese Singaporeans [corrected]. *European journal of nutrition* **2012**, *51*, 1033-40, doi:10.1007/s00394-011-0276-2.
29. Grantham, N.M.; Magliano, D.J.; Hodge, A.; Jowett, J.; Meikle, P.; Shaw, J.E. The association between dairy food intake and the incidence of diabetes in Australia: the Australian Diabetes Obesity and Lifestyle Study (AusDiab). *Public health nutrition* **2013**, *16*, 339-45, doi:10.1017/S1368980012001310.
30. von Ruesten, A.; Feller, S.; Bergmann, M.M.; Boeing, H. Diet and risk of chronic diseases: results from the first 8 years of follow-up in the EPIC-Potsdam study. *European journal of clinical nutrition* **2013**, *67*, 412-9,

doi:10.1038/ejcn.2013.7.

31. Pan, A.; Sun, Q.; Bernstein, A.M.; Schulze, M.B.; Manson, J.E.; Willett, W.C.; Hu, F.B. Red meat consumption and risk of type 2 diabetes: 3 cohorts of US adults and an updated meta-analysis. *The American journal of clinical nutrition* **2011**, *94*, 1088-96, doi:10.3945/ajcn.111.018978.

32. Steinbrecher, A.; Erber, E.; Grandinetti, A.; Kolonel, L.N.; Maskarinec, G. Meat consumption and risk of type 2 diabetes: the Multiethnic Cohort. *Public health nutrition* **2011**, *14*, 568-74, doi:10.1017/S1368980010002004.

33. Nanri, A.; Mizoue, T.; Noda, M.; Takahashi, Y.; Matsushita, Y.; Poudel-Tandukar, K.; Kato, M.; Oba, S.; Inoue, M.; Tsugane, S., et al. Fish intake and type 2 diabetes in Japanese men and women: the Japan Public Health Center-based Prospective Study. *The American journal of clinical nutrition* **2011**, *94*, 884-91, doi:10.3945/ajcn.111.012252.

34. Villegas, R.; Xiang, Y.B.; Elasy, T.; Li, H.L.; Yang, G.; Cai, H.; Ye, F.; Gao, Y.T.; Shyr, Y.; Zheng, W., et al. Fish, shellfish, and long-chain n-3 fatty acid consumption and risk of incident type 2 diabetes in middle-aged Chinese men and women. *The American journal of clinical nutrition* **2011**, *94*, 543-51, doi:10.3945/ajcn.111.013193.

35. Morimoto, Y.; Steinbrecher, A.; Kolonel, L.N.; Maskarinec, G. Soy consumption is not protective against diabetes in Hawaii: the Multiethnic Cohort. *European journal of clinical nutrition* **2011**, *65*, 279-82,

doi:10.1038/ejcn.2010.228.

36. Nanri, A.; Mizoue, T.; Takahashi, Y.; Kirii, K.; Inoue, M.; Noda, M.; Tsugane, S. Soy product and isoflavone intakes are associated with a lower risk of type 2 diabetes in overweight Japanese women. *The Journal of nutrition* **2010**, *140*, 580-6, doi:10.3945/jn.109.116020.

37. Djousse, L.; Gaziano, J.M.; Buring, J.E.; Lee, I.M. Dietary omega-3 fatty acids and fish consumption and risk of type 2 diabetes. *The American journal of clinical nutrition* **2011**, *93*, 143-50, doi:10.3945/ajcn.110.005603.

38. Mannisto, S.; Kontto, J.; Kataja-Tuomola, M.; Albanes, D.; Virtamo, J. High processed meat consumption is a risk factor of type 2 diabetes in the Alpha-Tocopherol, Beta-Carotene Cancer Prevention study. *The British journal of nutrition* **2010**, *103*, 1817-22, doi:10.1017/S0007114510000073.

39. Sluijs, I.; Beulens, J.W.; van der, A.D.; Spijkerman, A.M.; Grobbee, D.E.; van der Schouw, Y.T. Dietary intake of total, animal, and vegetable protein and risk of type 2 diabetes in the European Prospective Investigation into Cancer and Nutrition (EPIC)-NL study. *Diabetes care* **2010**, *33*, 43-8, doi:10.2337/dc09-1321.

40. Kirii, K.; Mizoue, T.; Iso, H.; Takahashi, Y.; Kato, M.; Inoue, M.; Noda, M.; Tsugane, S.; Japan Public Health Center-based Prospective Study, G. Calcium, vitamin D and dairy intake in relation to type 2 diabetes risk in a Japanese cohort. *Diabetologia* **2009**, *52*, 2542-50, doi:10.1007/s00125-009-1554-x.

41. Patel, P.S.; Sharp, S.J.; Luben, R.N.; Khaw, K.T.; Bingham, S.A.; Wareham, N.J.; Forouhi, N.G. Association between type of dietary fish and seafood intake and the risk of incident type 2 diabetes: the European prospective investigation of cancer (EPIC)-Norfolk cohort study. *Diabetes care* **2009**, *32*, 1857-63, doi:10.2337/dc09-0116.

42. van Woudenbergh, G.J.; van Ballegooijen, A.J.; Kuijsten, A.; Sijbrands, E.J.; van Rooij, F.J.; Geleijnse, J.M.; Hofman, A.; Witteman, J.C.; Feskens, E.J. Eating fish and risk of type 2 diabetes: A population-based, prospective follow-up study. *Diabetes care* **2009**, *32*, 2021-6, doi:10.2337/dc09-1042.

43. Kaushik, M.; Mozaffarian, D.; Spiegelman, D.; Manson, J.E.; Willett, W.C.; Hu, F.B. Long-chain omega-3 fatty acids, fish intake, and the risk of type 2 diabetes mellitus. *The American journal of clinical nutrition* **2009**, *90*, 613-20, doi:10.3945/ajcn.2008.27424.

44. Vang, A.; Singh, P.N.; Lee, J.W.; Haddad, E.H.; Brinegar, C.H. Meats, processed meats, obesity, weight gain and occurrence of diabetes among adults: findings from Adventist Health Studies. *Annals of nutrition & metabolism* **2008**, *52*, 96-104, doi:10.1159/000121365.

45. Villegas, R.; Gao, Y.T.; Yang, G.; Li, H.L.; Elasy, T.A.; Zheng, W.; Shu, X.O. Legume and soy food intake and the incidence of type 2 diabetes in the Shanghai Women's Health Study. *The American journal of clinical nutrition* **2008**, *87*, 162-7, doi:10.1093/ajcn/87.1.162.

46. Liu, S.; Choi, H.K.; Ford, E.; Song, Y.; Klevak, A.; Buring, J.E.; Manson, J.E. A prospective study of dairy intake and the risk of type 2 diabetes in women. *Diabetes care* **2006**, *29*, 1579-84, doi:10.2337/dc06-0256.
47. Choi, H.K.; Willett, W.C.; Stampfer, M.J.; Rimm, E.; Hu, F.B. Dairy consumption and risk of type 2 diabetes mellitus in men: a prospective study. *Archives of internal medicine* **2005**, *165*, 997-1003, doi:10.1001/archinte.165.9.997.
48. Zazpe, I.; Beunza, J.J.; Bes-Rastrollo, M.; Basterra-Gortari, F.J.; Mari-Sanchis, A.; Martinez-Gonzalez, M.A.; Investigators, S.U.N.P. Egg consumption and risk of type 2 diabetes in a Mediterranean cohort; the sun project. *Nutricion hospitalaria* **2013**, *28*, 105-11, doi:10.3305/nh.2013.28.1.6124.
49. Kurotani, K.; Nanri, A.; Goto, A.; Mizoue, T.; Noda, M.; Oba, S.; Sawada, N.; Tsugane, S.; Japan Public Health Center-based Prospective Study, G. Cholesterol and egg intakes and the risk of type 2 diabetes: the Japan Public Health Center-based Prospective Study. *The British journal of nutrition* **2014**, *112*, 1636-43, doi:10.1017/S000711451400258X.
50. Djousse, L.; Petrone, A.B.; Hickson, D.A.; Talegawkar, S.A.; Dubbert, P.M.; Taylor, H.; Tucker, K.L. Egg consumption and risk of type 2 diabetes among African Americans: The Jackson Heart Study. *Clinical nutrition* **2016**, *35*, 679-84, doi:10.1016/j.clnu.2015.04.016.
51. Guo, J.; Hobbs, D.A.; Cockcroft, J.R.; Elwood, P.C.; Pickering, J.E.;

Lovegrove, J.A.; Givens, D.I. Association between egg consumption and cardiovascular disease events, diabetes and all-cause mortality. *European journal of nutrition* **2018**, *57*, 2943-2952, doi:10.1007/s00394-017-1566-0.

52. Djousse, L.; Kamineni, A.; Nelson, T.L.; Carnethon, M.; Mozaffarian, D.; Siscovick, D.; Mukamal, K.J. Egg consumption and risk of type 2 diabetes in older adults. *The American journal of clinical nutrition* **2010**, *92*, 422-7, doi:10.3945/ajcn.2010.29406.
53. Djoussé, L.; Michael Gaziano, J.; Buring, J.E.; Lee, I.M. Egg consumption and risk of type 2 diabetes in men and women. *Diabetes Care* **2009**, *32*, 295-300, doi:10.2337/dc08-1271.
54. Wallin, A.; Forouhi, N.G.; Wolk, A.; Larsson, S.C. Egg consumption and risk of type 2 diabetes: a prospective study and dose-response meta-analysis. *Diabetologia* **2016**, *59*, 1204-13, doi:10.1007/s00125-016-3923-6.
55. Ericson, U.; Hellstrand, S.; Brunkwall, L.; Schulz, C.A.; Sonestedt, E.; Wallstrom, P.; Gullberg, B.; Wirfalt, E.; Orho-Melander, M. Food sources of fat may clarify the inconsistent role of dietary fat intake for incidence of type 2 diabetes. *The American journal of clinical nutrition* **2015**, *101*, 1065-80, doi:10.3945/ajcn.114.103010.
56. Montonen, J.; Jarvinen, R.; Heliovaara, M.; Reunanen, A.; Aromaa, A.; Knekt, P. Food consumption and the incidence of type II diabetes mellitus. *European journal of clinical nutrition* **2005**, *59*, 441-8, doi:10.1038/sj.ejcn.1602094.

57. Villegas, R.; Shu, X.O.; Gao, Y.T.; Yang, G.; Cai, H.; Li, H.; Zheng, W. The association of meat intake and the risk of type 2 diabetes may be modified by body weight. *International journal of medical sciences* **2006**, *3*, 152-9, doi:10.7150/ijms.3.152.

58. Song, Y.; Manson, J.E.; Buring, J.E.; Liu, S. A prospective study of red meat consumption and type 2 diabetes in middle-aged and elderly women: the women's health study. *Diabetes care* **2004**, *27*, 2108-15, doi:10.2337/diacare.27.9.2108.

59. van Dam, R.M.; Willett, W.C.; Rimm, E.B.; Stampfer, M.J.; Hu, F.B. Dietary fat and meat intake in relation to risk of type 2 diabetes in men. *Diabetes care* **2002**, *25*, 417-24, doi:10.2337/diacare.25.3.417.

60. Lajous, M.; Bijon, A.; Fagherazzi, G.; Balkau, B.; Boutron-Ruault, M.C.; Clavel-Chapelon, F. Egg and cholesterol intake and incident type 2 diabetes among French women. *The British journal of nutrition* **2015**, *114*, 1667-73, doi:10.1017/S0007114515003190.
